# AI-based modeling of CO_2_ footprint in geopolymer concrete production containing GGBFS as a by-product from the iron industry

**DOI:** 10.1038/s41598-025-27361-7

**Published:** 2025-12-08

**Authors:** Ramin Kazemi, Ali Bashtani, Seyedali Mirjalili

**Affiliations:** 1 Independent Researcher, Sabzevar, Iran; 2 Independent Researcher, Sabzevar, Iran; 3 Centre for Artificial Intelligence Research and Optimisation, Torrens University Australia, Brisbane, Australia; 4 Department of Computer Science, VSB-Technical University of Ostrava, Ostrava, Czech Republic; 5 University Research and Innovation Center, Obuda University, Budapest, Hungary

**Keywords:** CO_2_ footprint, Geopolymer concrete, Ground granulated blast-furnace slag, Artificial intelligence, Compressive strength, Optimization, Artificial Intelligence, Algorithm, Carbon emissions., Engineering, Environmental sciences

## Abstract

This study investigates the CO_2_ footprint (CF) of geopolymer concrete (GPC) produced using ground granulated blast-furnace slag (GGBFS), an industrial by-product and alternative to ordinary Portland cement. Given the diversity of influential variables and the necessity for repeated testing due to changing materials and conditions, an artificial intelligence (AI)-based framework is considered an effective strategy to diminish reliance on experimental work. A novel hybrid model combining artificial neural network and biogeography-based optimization (ANN-BBO) is developed to predict the CF associated with GGBFS-based GPC production. The model incorporates 25 key variables related to precursor content and its chemical composition, activator content and characteristics, aggregate content, mix design, and curing conditions. Through rigorous evaluation using statistical metrics, error histograms, and *k*-fold cross-validation, the proposed ANN-BBO model demonstrated high accuracy, with 89% of its predictions falling within a 5% error margin compared to 62% for the single ANN, indicating a 27% improvement in prediction accuracy. Sensitivity analysis enhanced interpretability by identifying the superplasticizer and coarse aggregate as the key positive and negative contributors to the CF, while also suggesting that 400 kg/m^3^ of GGBFS is the ideal content for maximizing compressive strength and minimizing carbon emissions in GGBFS-based GPC production.

**Supplementary Information:**

The online version contains supplementary material available at 10.1038/s41598-025-27361-7.

## Introduction

### Background

**Fig. 1:**
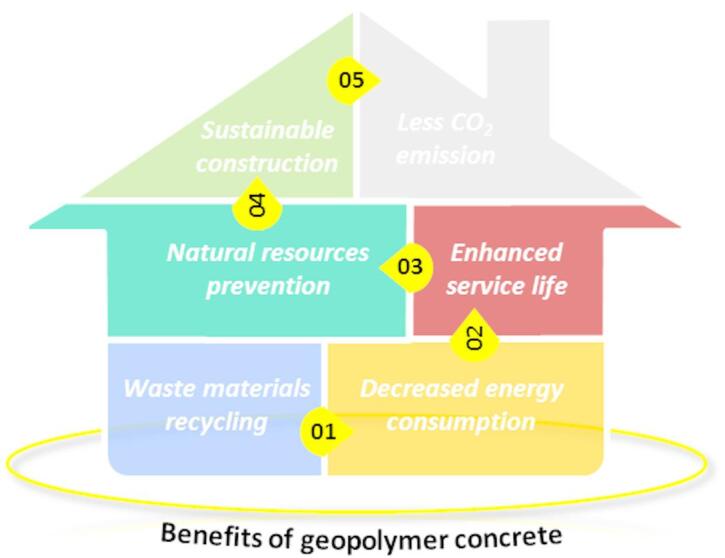
Benefits of utilizing geopolymer concrete in the construction industry.

The production of ordinary Portland cement (OPC), the main ingredient in concrete preparation, contributes approximately 7% of global CO_2_ emissions^1^. To minimize the CO_2_ footprint (CF) from OPC production and promote the development of sustainable and eco-friendly concrete, researchers have focused on partially/fully substituting OPC with replacement materials known as supplementary cementitious materials (SCMs), such as agro-industrial ash (e.g. fly ash and rice husk ash) and industrial by-products (e.g. ground granulated blast-furnace slag (GGBFS) and silica fume)^2,3^. Utilizing these materials enhances both the strength and rheological characteristics of concrete while also lowering CO_2_ emissions and minimizing environmental impact^4^. Concretes made from the reaction of OPC-replaced SCMs containing aluminate- and silicate-rich compounds as precursors with a caustic activator are called geopolymer concretes (GPCs). Figure 1 highlights the benefits of utilizing GPC in the construction industry.

### GGBFS and GPC production

**Fig. 2:**
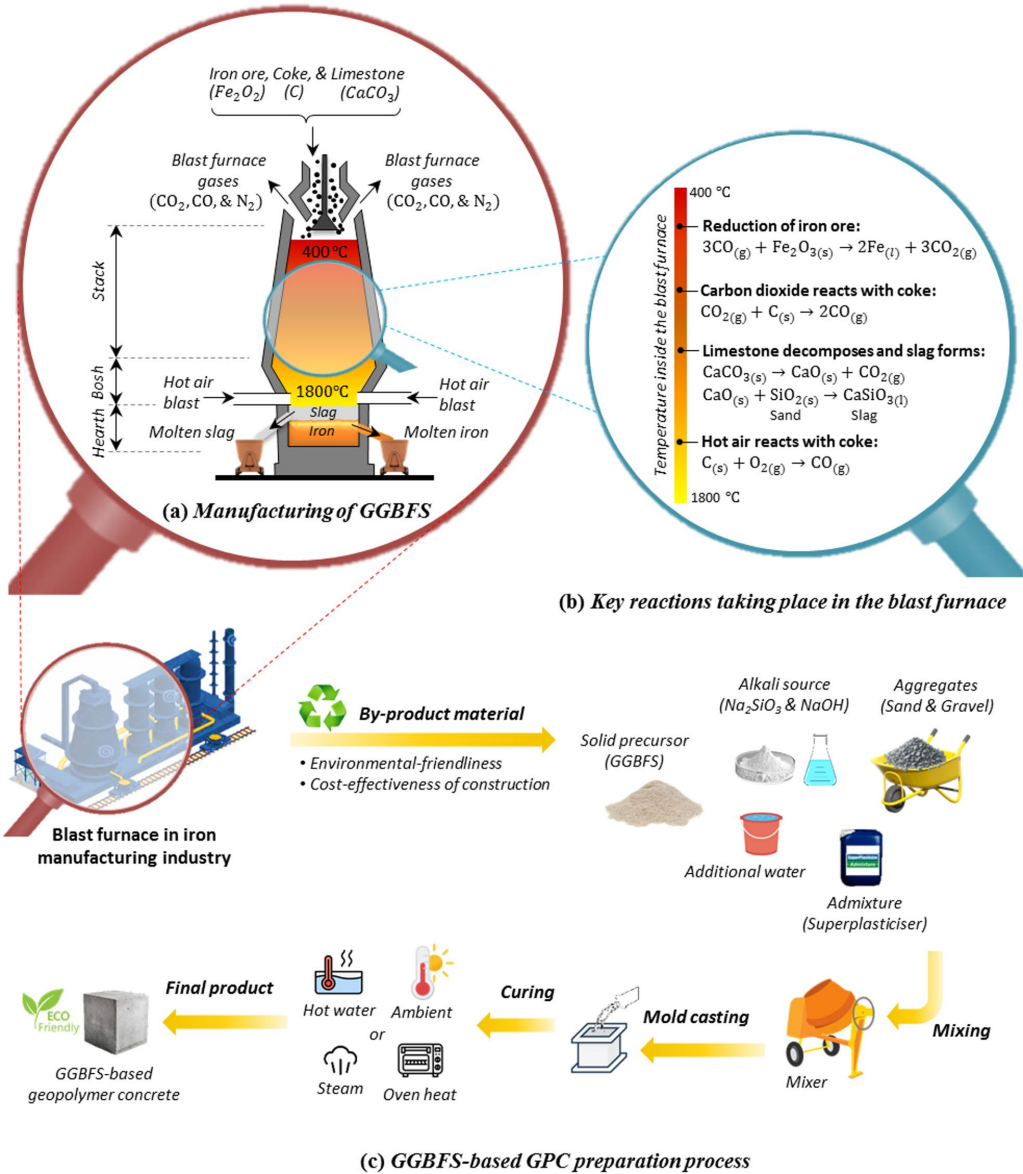
An in-depth overview of GGBFS and GGBFS-based GPC production process: (**a**) GGBFS as a by-product during the iron production process, (**b**) Details of the key reactions taking place in the blast furnace, and (**c**) GGBFS-based GPC preparation.

One of the most widely used precursors in the production of geopolymer concrete (GPC) is ground granulated blast furnace slag (GGBFS), an industrial by-product from the iron-making industry. Blast furnaces operate at around 1500 °C and process a controlled mixture of iron ore (Fe_2_O_3_), coke (C), and limestone (CaCO_3_). During this process, iron ore is reduced to molten iron, while the remaining materials form a slag that floats on the surface (see Fig. 2a,b). This molten slag is tapped off and undergoes three main steps to become GGBFS: (i) rapid quenching using air, water, or water showers; (ii) draining and drying during storage, during which it gains cementitious properties through chemical reactions with water; and (iii) grinding to the required fineness^5^. Each tonne of pig iron yields about 300 kg of slag, mainly composed of calcium oxide (30–50%), silica (30–40%), and alumina (7–18%)—all typical components in cementitious materials^6^.

The use of GGBFS in concrete provides several significant benefits over conventional concrete. It helps mitigate thermal cracking by reducing the heat of hydration. Additionally, it enhances workability, making placement and compaction easier. GGBFS also improves durability by increasing resistance to sulfate attack and chloride penetration, which reduces the risk of steel reinforcement corrosion. Furthermore, it minimizes the occurrence of alkali-silica reaction, a damaging internal chemical reaction that leads to cracking and deterioration of concrete structures^6–8^.

Due to these advantages, GGBFS is used not only in GPC but also in precast concrete, wastewater infrastructure (e.g., treatment plants and sewer pipes), marine structures (e.g., ports and piers), and cement-based products like tiles and blocks. The production of GGBFS-based GPC, shown in Fig. 2c, involves using GGBFS as a binder rich in aluminosilicates, activated by high-pH alkaline solutions (Na_2_SiO_3_ and NaOH), along with aggregates and water. These mixtures are typically cured under various conditions.

Research indicates that GGBFS-based GPC is viewed as a sustainable substitute for OPC concrete, offering the potential to substantially lower CO_2_ release in the construction sector^9,10^. In this regard, some studies^11^ have indicated a reduction in CO_2_ emissions ranging from 20 to 30% compared to OPC-based concrete. Additionally, a study by Crossin^12^ found that substituting cement with GGBFS in concrete could lower CO_2_ emissions by 47.5%. GGBFS-based GPC is achieved during a polymerization process between GGBFS and alkaline solutions, while this activation process causes the release of CO_2_. This process is affected by alkaline solutions^13^ and curing conditions^14^, which complexly interact with SCMs. In other words, this variety in reported CO_2_ emission can be associated with several factors such as the composition of raw SCMs, the type and concentration of alkaline solutions, admixture, and curing time and temperature. These factors contribute notably to the CF of GGBFS-based GPC. However, it is important to recognize that conventional laboratory methods for preparing and testing to determine the optimum values of these factors and their influence on the CF of GGBFS-based GPC production are both time-consuming and expensive. Besides, any changes in compositions or conditions require additional testing. Bearing this in mind, the time has come to move towards an artificial intelligence (AI)-based framework as a viable and effective strategy to reduce reliance on experimental activities.

### Application of AI in GPC

**Fig. 3:**
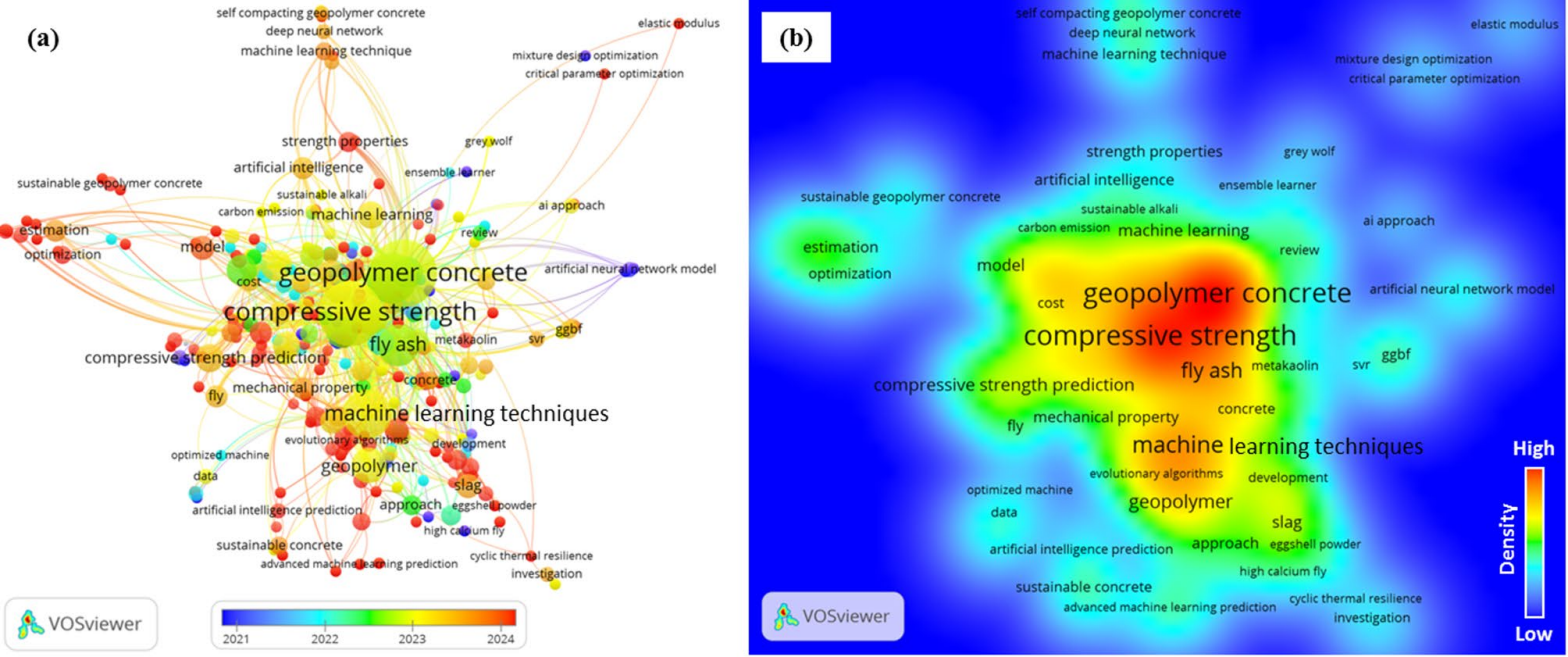
(**a**) Scientometric overview and (**b**) density visualization of AI application in GPC.

As in other fields, the development of AI in civil engineering—particularly in various areas of concrete technology^15^, such as mix design^16,17^, durability^18^, strength properties^19,20^, and sustainable concrete^21^—has attracted the attention of researchers in recent years. For better visualization, we utilized the VOSviewer tool^22^ to present a scientometric overview and density visualization based on the co-occurrence of key title words from publications in the field of AI techniques applied to GPC, as illustrated in Fig. 3. By scrutinizing AI studies on GGBFS-based GPC, it was found that most of them have focused on predicting strength properties, especially compressive strength^23–28^.

This is despite the fact that, to the best of the authors’ knowledge, the use of AI prediction models for the CF of GGBFS-based GPC is only limited to a recent study by Al-Fakih et al.^29^ in 2024. The best performance of their proposed model is reported with a coefficient of determination of 0.95. However, a close examination of the dataset used in^29^ for modeling revealed that 7% of it contained duplicate data. These duplicates should not be overlooked, as they can create major problems such as overemphasis, biased predictions, distorted feature importance, and, in a word, mask the real performance of the model. Hence, it needs to be addressed. Besides, the structure of AI models has increasingly evolved by integrating meta-heuristic optimization methods with predictive models. This integrated technique leverages the strengths of individual prediction models while mitigating their weaknesses by identifying efficient solutions and enhancing accuracy. As a result, it can lead to more dependable results. Given this, further research in this direction is highly valuable for confirming its general applicability and effectiveness.

## Research significance

The importance of this research is that it is aligned with goals 12 and 13 of the United Nations’ 17 sustainable development goals (SDGs), which focus on promoting sustainable production by minimizing waste through the use of by-products and material recycling (SDG 12), and combating climate change by investing in low-carbon development (SDG 13). With this in mind, the present study aims to develop a novel hybrid AI-based framework called artificial neural network integrated with biogeography-based optimization (ANN-BBO) to model the CF associated with producing GPC in which incorporates GGBFS derived from by-products of the iron-making industry as a cement substitute. AI-based approaches are able to model various features to achieve the defined goal while overcoming the limitations of traditional laboratory methods, such as being labor-intensive and costly due to the need for additional testing under changing conditions and materials. In the following, the paper is structured into six sections. Section 3 details the collection and description of the database, while Sect. 4 provides an overview of AI-based techniques used. Section 5 focuses on model development, followed by Sect. 6, which presents the results and discussion. Section 7 addresses the limitations and suggests directions for future research, and Sect. 8 wraps up with a summary of the key findings.

## Database and data description

### Data acquisition

In this study, the CF prediction of GGBFS-based GPC is evaluated by tracking the amount of CO_2_ emissions per material involved in its construction. In this approach, all the materials involved in producing GGBFS-based GPC, along with their respective quantities, are considered inputs, while the CF is treated as output. For this purpose, a comprehensive database was compiled from the literature. This database includes 122 data records extracted from 19 published studies and was utilized as the foundation for developing AI-based models (see Table 1). Full details of this dataset are available in the supplementary data. It is worth noting that the impact of the CF resulting from transportation between the extraction/manufacturing of raw materials and the batching plant for the production of GGBFS-based GPC is not considered due to the lack of data in this field.

**Table 1:** Details of the extracted dataset.

No. ID	Authors [Ref.]	Year	No. data	Precursor content of GGBFS (kg/m^3^)
1	Adam^30^	2004	3	412–419
2	Bernal, et al.^31^	2012	3	400
3	Chi^32^	2012	6	424
4	Kar, et al.^33^	2013	6	340–400
5	Parthiban and Saravana^34^	2014	12	356.36
6	Wardhono^35^	2014	1	415
7	Behfarnia, et al.^36^	2015	9	336–428.60
8	Parthiban and Vaithianathan^37^	2015	3	400
9	Ramani and Chinnaraj^38^	2015	1	394
10	Emmanuel^39^	2016	4	394.29
11	Rajarajeswari and Dhinakaran^40^	2016	27	560
12	Venkatesan and Pazhani^41^	2016	3	394
13	Hadi, et al.^42^	2017	9	400–450
14	Takekar and Patail^43^	2017	2	405
15	Wardhono, et al.^44^	2017	1	415
16	Ding, et al.^45^	2018	1	400
17	Mehta and Siddique^46^	2018	1	394
18	Oyebisi, et al.^47^	2018	3	390
19	Aliabdo, et al.^48^	2019	27	400

### CO_2_ footprint calculation

**Fig. 4:**
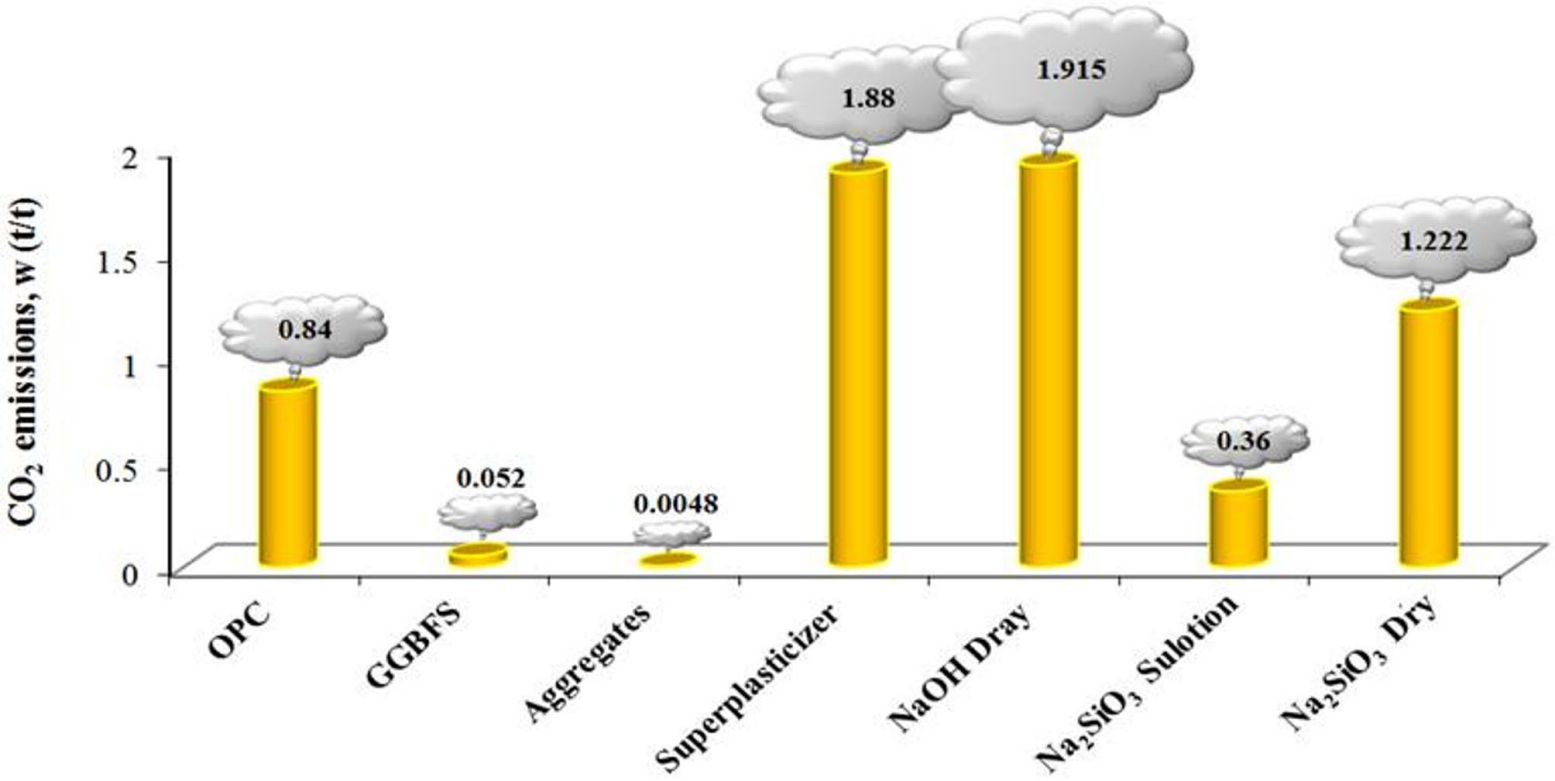
CO_2_ emissions in tons for the production of each ton of constituent material to the construction of GGBFS-based GPC^50^.

To measure the CF, the effect of two main factors on CO_2_ emissions from the manufacture of GGBFS-based GPC, including those resulting from the production/extraction of constituent materials and the oven curing temperature, is considered. To calculate these factors, the equation proposed in 2023^49^ was used.

1 $$\:{CO}_{2\:}emission\:\left(\frac{t}{{m}^{3}}\right)=\:\sum\:_{i}{w}_{i}.\:{m}_{i}+\left(0.6417.\:T-16.0417\right).t$$

where *w*_*i*_ represents the amount of CO_2_ emitted per ton for the production of one ton of each constituent material. Figure 4 illustrates CO_2_ emissions contribution of each constituent, as reported in^50^. *m*_*i*_ represents the mass of each constituent material per *t/m*^*3*^ of fresh mix. *T* and *t* are the curing temperature and time in °C and days, respectively. Note that *t* is only included in the calculations if the *T* exceeds 25 °C, otherwise it is assigned a value of zero.

The equation indicates a reasonable level of accuracy in estimating CF for mixtures drawing on the datasets available in^50,51^. This simplified mathematical model, however, overlooks factors such as the effects of material transportation and the variations between the settings in mass production sites and laboratory-controlled conditions due to the lack of data.

### Data visualization plots

**Fig. 5:**
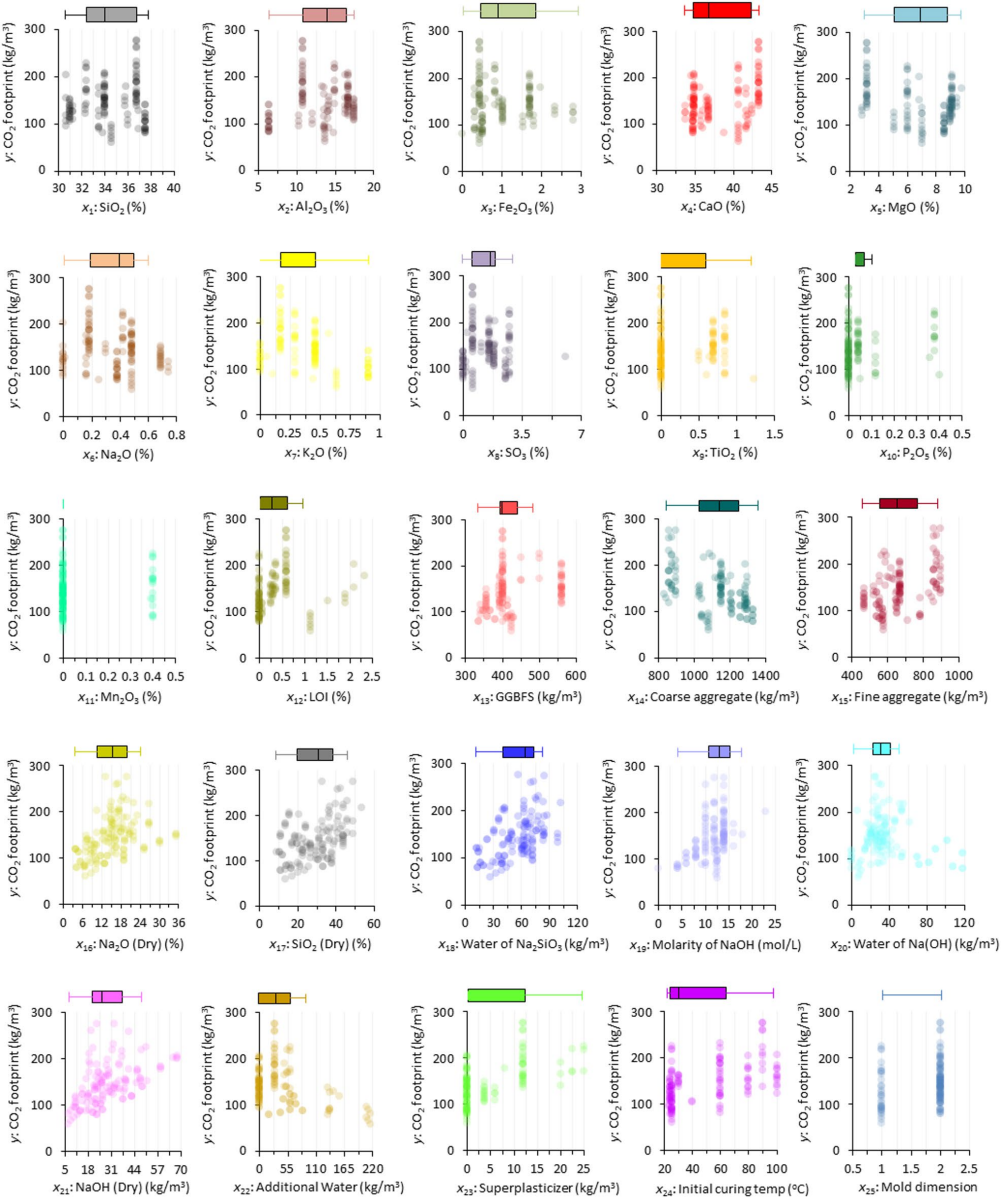
The scatter and box plots of features.

To begin, the first step is to introduce the variables considered and perform a descriptive analysis of them in order to evaluate their quantity and quality. The rationale behind the selection of input variables was according to their potential influence on CO_2_ emissions in GGBFS-based GPC production and their availability to create an extensive database. To ensure the integrity and reliability of the dataset, a comprehensive data preprocessing procedure was implemented. Only samples containing complete records for all input and output parameters were retained. Studies lacking essential metadata or employing non-standard testing protocols were excluded. Furthermore, duplicate entries were identified and removed by cross-referencing sample identifiers and test conditions. Samples with missing critical parameters were also systematically eliminated to maintain consistency and analytical robustness. The input variables considered for the prediction models included the main chemical composition (*x*_1_-*x*_12_), precursor content (*x*_13_), aggregates content (*x*_14_-*x*_15_), Na_2_SiO_3_ composition (*x*_16_-*x*_18_), Na(OH) composition (*x*_19_-*x*_21_), mix design and curing conditions (*x*_22_-*x*_25_). The CF value (*y*) of GGBFS-based GPC is considered as the output variable in the prediction models. Figure 5 illustrates the distribution of data points between the input (*x*_1_-*x*_25_) and the output (*y*) variables. By carefully examining the plots belonging to each variable, it can be found that the CF shows the most sensitivity by changing the content of which variables. Although the *x*_23_ (superplasticizer) is not used in most GGBFS-based GPC designs and has zero content, it has significantly increased the CF in the designs where it has been used. The next variable is the *x*_24_ (initial curing temperature), which plays a significant role in contributing to the CF. The clustering of data points around the T_c_ of 25 °C and 60 °C indicates that these temperatures are commonly used for curing GGBFS-based GPC designs. Nevertheless, a significant trend in the data distribution plot is the apparent rise in the CF as the *x*_24_ increases. NaOH (Dry) (*x*_21_), which is used as the alkaline source, is the third variable affecting the CF in GGBFS-based GPC designs. Its plot indicates that values used up to 10 (kg/m^3^) show a very low CF, and exceeding this value significantly increases the CF. Scrutinizing these variables is important because using less SP and NaOH (Dry) and curing at low temperatures in GGBFS-based GPC designs can be an effective approach for reducing the CF.

Table 2 encapsulates a comprehensive summary of key statistical measures. These metrics provide a comprehensive outlook of the dataset by showcasing each variable’s central tendencies and variability, thereby offering valuable understanding of the overall data features.

**Table 2:** Descriptive statistics of the features.

ID No.: Variables	Role	Type	Unit	Range (Min–Max)	Median	Mean	Standard deviation	Kurtosis	Skewness
*x*_1_: SiO_2_	The main chemical compositions	Input	%	30.60–37.50	34.01	34.25	2.23	− 1.26	− 0.13
*x*_2_: Al_2_O_3_		Input	%	6.40–17.41	14.11	13.71	3.19	− 0.35	− 0.72
*x*_3_: Fe_2_O_3_		Input	%	0–2.80	0.83	1.02	0.67	− 0.28	0.78
*x*_4_: CaO		Input	%	33.75–43.34	36.77	38.52	3.58	− 1.70	0.21
*x*_5_: MgO		Input	%	2.87–9.76	7.05	6.74	2.39	− 1.38	− 0.43
*x*_6_: Na_2_O		Input	%	0–0.74	0.40	0.35	0.21	− 0.95	0.00
*x*_7_: K_2_O		Input	%	0–0.90	0.29	0.34	0.24	0.01	0.55
*x*_8_: SO_3_		Input	%	0–6.08	1.55	1.39	0.99	2.53	0.87
*x*_9_: TiO_2_		Input	%	0–1.23	0.00	0.30	0.37	− 1.49	0.54
*x*_10_: P_2_O_5_		Input	%	0–0.40	0.00	0.05	0.11	5.12	2.54
*x*_11_: Mn_2_O_3_		Input	%	0–0.40	0.00	0.06	0.14	2.49	2.11
*x*_12_: LOI		Input	%	0–2.32	0.28	0.38	0.50	4.32	2.02
*x*_13_: GGBFS	Precursor content	Input	kg/m^3^	336.00–560.00	400.00	433.14	72.69	− 0.63	0.94
*x*_14_: Coarse aggregate	Aggregate content	Input	kg/m^3^	832.00–1327.20	1148.00	1101.03	151.28	− 1.10	− 0.40
*x*_15_: Fine aggregate		Input	kg/m^3^	467.12–894.00	668.00	676.34	132.78	− 0.98	0.29
*x*_16_: Na_2_O (Dry)	Na_2_SiO_3_ composition	Input	kg/m^3^	3.78–35.18	15.44	15.86	6.58	0.62	0.62
*x*_17_: SiO_2_ (Dry)		Input	kg/m^3^	9.07–53.80	31.14	29.79	11.01	− 1.08	− 0.06
*x*_18_: Water		Input	kg/m^3^	12.10–102.30	63.00	58.54	19.87	− 0.35	− 0.33
*x*_19_: Molar concentration of NaOH	Na(OH) composition	Input	mol/L	0–23.00	12.48	11.89	2.98	3.05	− 0.72
*x*_20_: Water		Input	kg/m^3^	0–117.95	28.94	32.20	21.48	4.90	1.81
*x*_21_: NaOH (Dry)		Input	kg/m^3^	6.53–67.76	25.66	28.62	13.65	0.62	0.86
*x*_22_: Additional W	Mix design and curing conditions	Input	kg/m^3^	0–216.00	30.00	37.75	54.85	3.17	1.92
*x*_23_: Superplasticizer		Input	kg/m^3^	0–25	0.00	4.93	6.94	0.74	1.29
*x*_24_: Initial curing temp		Input	°C	23–100	30.00	49.95	27.76	− 1.24	0.55
*x*_25_: Mold dimension		Input	–	1–2	1.00	1.25	0.43	− 0.58	1.19
*y*: CO_2_ footprint		Output	kg/m^3^	59.23–276.11	142.52	147.43	44.03	0.36	0.60

**Fig. 6:**
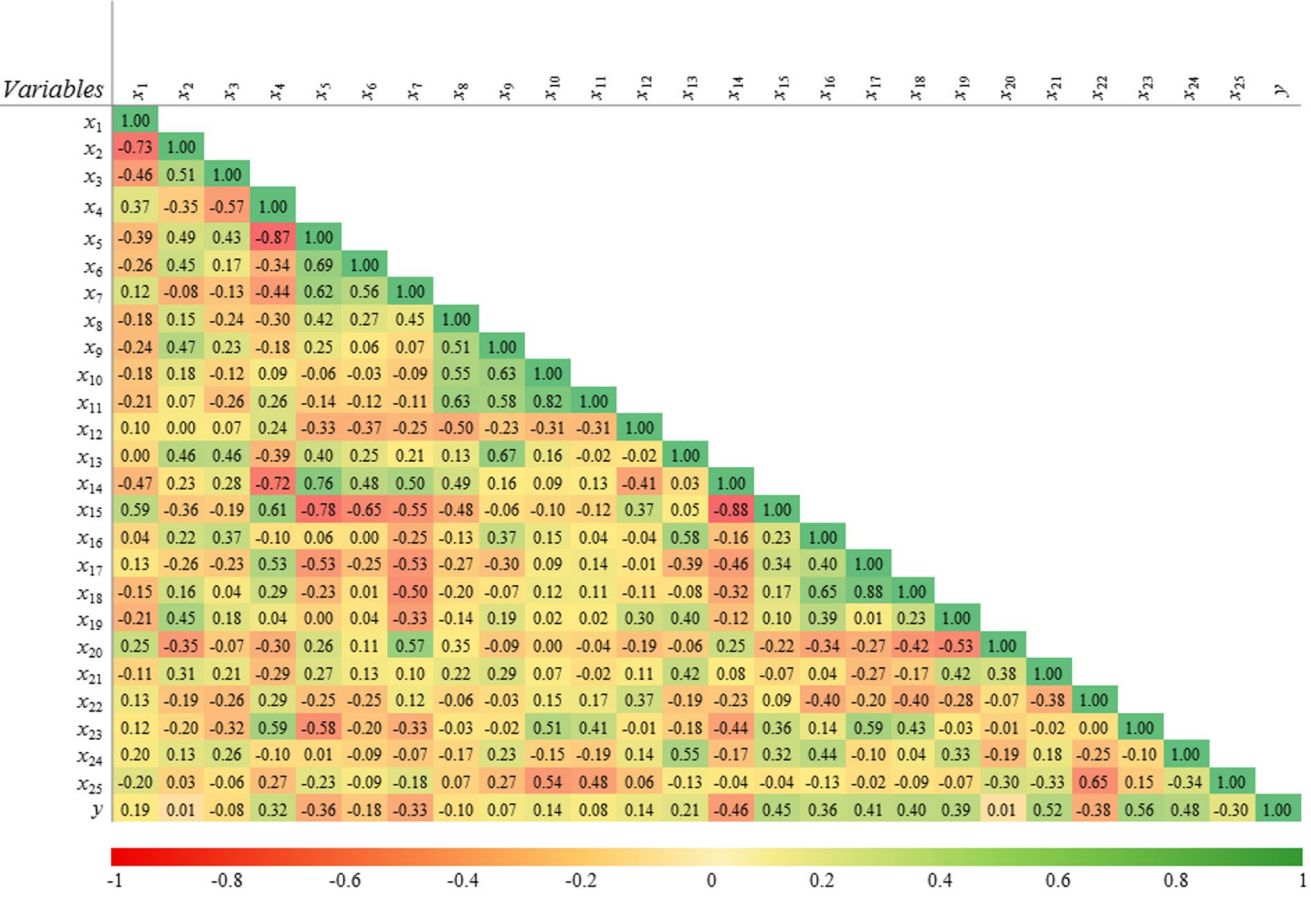
The correlation heatmap of features.

To further analyse the data, a correlation heatmap is employed to visually represent the pairwise correlation between variables, as shown in Fig. 6. This heatmap plot illustrates the effects of considering each variable on each other. In particular, the last row of the correlation matrix highlights a well-rounded view of the Pearson correlation coefficient for each pairwise correlation between the input and output variables. The Pearson correlation coefficient, typically represented by *r*, quantifies the linear relationship between two variables. Its values ​​are in the range [− 1, + 1], with the upper and lower limits indicating positive and negative correlations, respectively.

## AI-based modeling: an overview of techniques used

### Artificial neural network (ANN)

**Fig. 7:**
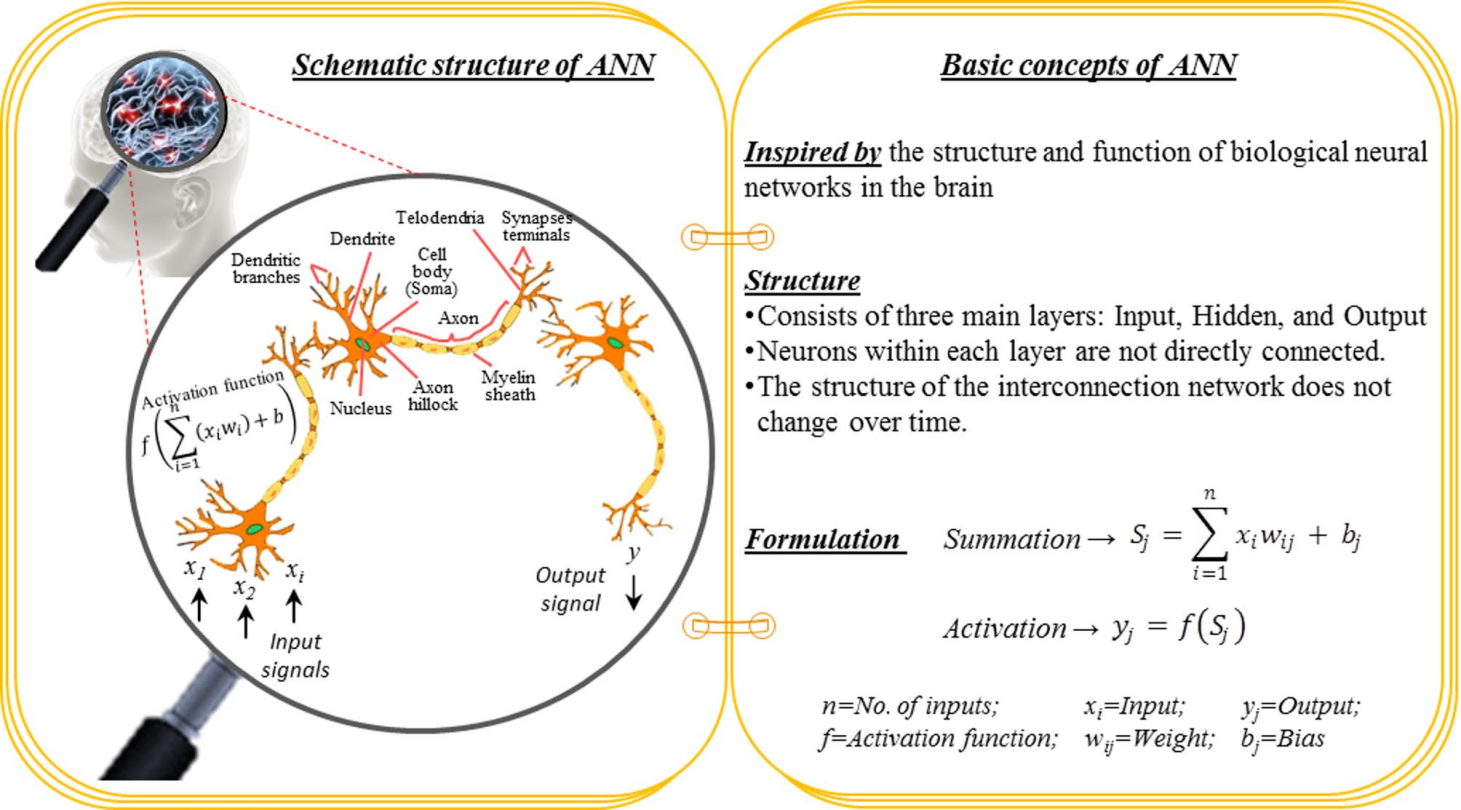
A concise overview of the ANN model.

The idea behind ANN is rooted in the intricate neural connections of the human brain. An ANN comprises interconnected neurons organized into three main layers: input, hidden, and output. Importantly, neurons within the same layer are not directly connected, maintaining a structured hierarchy. The input layer’s neurons are tailored to correspond with the number of inputs. In contrast, the output layer represents the network’s predicted results, reflecting the final outcomes of its processing. Among these layers, the hidden layers play a critical role in receiving, processing, and transmitting signals, mimicking biological neural processes. Signal processing occurs in a structured sequence: (i) receiving signals through dendrites, (ii) assigning weights to each input by an activation function, (iii) transmitting the signals through the axon, and finally, (iv) passing them to the next neuron via the axon terminals^52^. Activation functions are employed by neurons to process incoming signals (inputs) and pass them from one layer to the next^53^. Figure 7 illustrates a brief overview of the ANN model.

### Biogeography-based optimization (BBO)

**Fig. 8:**
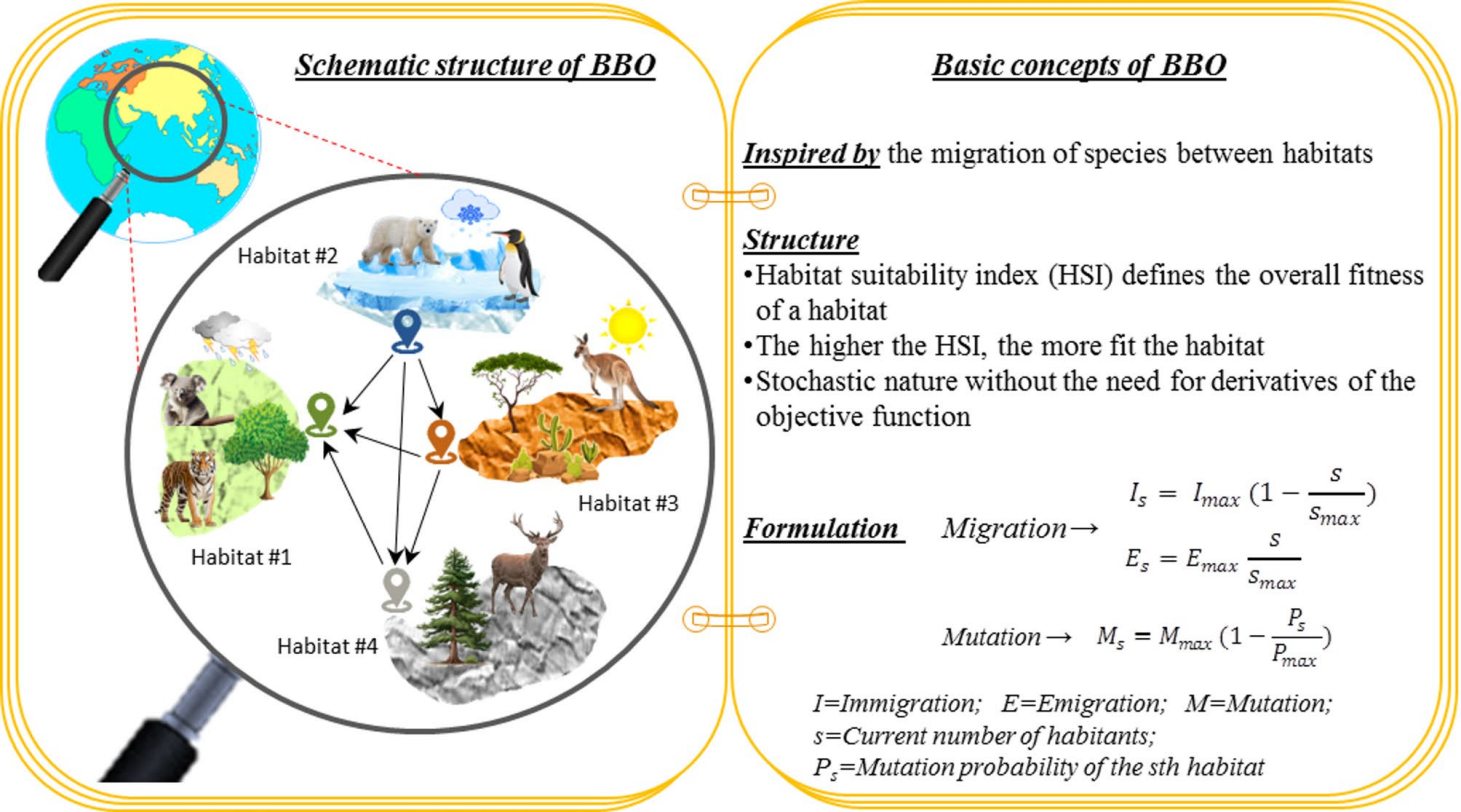
A concise overview of the BBO method.

The idea behind nature-inspired BBO optimization is based on the principles of biogeography, which draws insights from species’ migrations between various habitats. As a heuristic optimization approach, BBO mimics biological processes such as migration and mutation to tackle complex problems effectively^54^. One of the main aspects of BBO is inspired by species migration. In this algorithm, potential solutions are represented as habitats or candidate solutions. These habitats continuously exchange information and adapt to improve the overall quality of the population across iterations. Migration involves exchanging information, referred to as migration vectors, between various habitats. The likelihood of a solution migrating to a different habitat depends on suitability index variables (SIVs) and the habitat suitability index (HSI), which correspond to independent variables and the habitat’s overall fitness, respectively^55^. Another key aspect of BBO is mutation, which simulates adaptability in biological systems. By introducing random changes to solutions, the algorithm maintains diversity within the population, reducing the likelihood of early convergence to suboptimal results. It enables the investigation of a wider solution space and the discovery of potentially better outcomes. Solutions are assessed based on fitness, reflecting their effectiveness in addressing the problem. Those with higher fitness are prioritized for reproduction, while those with lower fitness are progressively discarded. This selection trend, akin to the principle of survival of the fittest, gradually steers the population toward better solutions during successive iterations. As a result, the BBO incrementally improves its population, resulting in progressively enriched outcomes. Figure 8 depicts a brief overview of the BBO method.

Investigating the strengths of the optimization method utilized in this research, BBO, is a worthwhile endeavor as it helps justify its superiority over other methods. In this regard, a widely referenced study^56^ has evaluated the performance of six well-known heuristic techniques, highlighting BBO’s distinctive ability to make significant changes in candidate solutions through migration and crossover techniques. This characteristic distinguishes BBO, as it helps avoid the common problem of getting stuck in local minima. Additionally, its stochastic nature removes the requirement for objective function derivatives, improving its effectiveness in addressing various real-world problems^57^.

## Development and evaluation of models

### Model construction using ANN and BBO

**Fig. 9:**
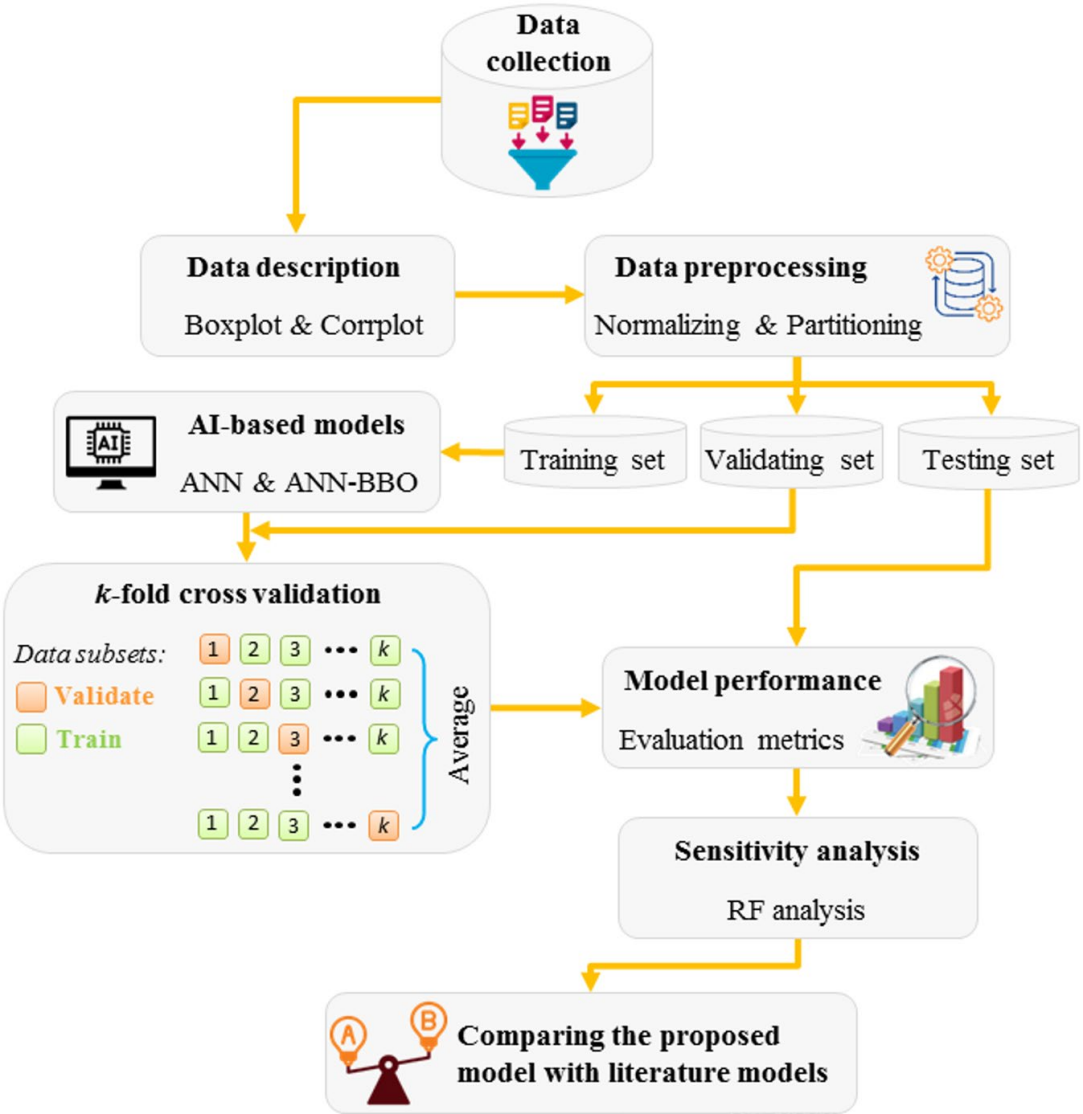
Methodology flowchart in the present research.

This study aims to develop an AI-based framework to model the CF in GGBFS-based GPC. To this end, two ANN and ANN-BBO models are proposed. These models offer valuable insights into the effectiveness of the hybrid version of the ANN compared to its single model. The BBO technique is applied to guide the predictive model toward achieving more accurate results with minimal error. Figure 9 illustrates the methodology flowchart in the present research. In this regard, data were gathered from relevant literature, as detailed in Sect. 3, covering a broad spectrum of information related to the objectives of the present study. During the data wrangling phase for the GGBFS-based GPC dataset, our primary objective was to prepare the data for predicting the CF in the production of GGBFS-based GPC. This dataset includes all the key features involved in the production process. As all the mix designs were derived from laboratory programs validated by previous studies, we ensured the consistency of the data. To promote that each variable contributes fairly during analysis and model training, the data is normalized by scaling numerical features to a standard range using the Z-score. This helps prevent negative impacts on model performance due to varying scales, bringing all data to a common scale. The Z-score normalization formula is as follows:

2 $$\:{Z-score}_{{data}_{i}}=\frac{{data}_{i}-\:{Mean}_{data}\:}{{Standard\:deviation}_{data}}$$

The next step involves dividing the dataset into three sets: train, validate, and test. These sets serve distinct purposes: the training set is used to train the model, the validating set is utilized to avoid overfitting, and the testing set assesses its generalizability performance. Numerous studies evaluating various learning algorithms have highlighted the exceptional performance of the Levenberg-Marquardt algorithm^58,59^, making it the chosen algorithm for this research. Additionally, the hyperbolic tangent function is used as the transfer function, in line with the recommendation in Haykin’s widely cited book^60^. The number of hidden layer/s and their respective nodes affect the convergence of the model’s learning process^61^. According to the universal approximation theorem^62^, the model is designed with one hidden layer. As a result, the initial structure of the model is in the form of *25*_Input_-*nodes*_Hidden_-*1*_Output_. The number of *nodes*_Hidden_ is associated with the number of inputs and outputs. Accordingly, subsection 6.1 (Hyperparameter tuning of models) will provide a well-rounded analysis to obtain the final structure of the model (i.e., the determination of hidden layer nodes).

### Performance metrics

Statistical metrics were employed to assess the consistency and precision of the suggested models, with the goal of developing an efficient model. Table 3 comprehensively summarizes the mathematical formula and ideal value for each metric used.

**Table 3:** Summary of statistical metrics used.

Metric Formula	Range Ideal value
*Mean absolute error (MAE)*	(0, $\:+\infty\:$) 0
$$\:MAE=\frac{1}{N}\sum\:_{\text{i}=1}^{N}\left|{{y}_{m}}_{i}-{{y}_{p}}_{i}\right|$$	
*Root mean squared error (RMSE)*	(0, $\:+\infty\:$) 0
$$\:RMSE=\sqrt{\frac{1}{N}\sum\:_{\text{i}=1}^{N}{({{y}_{m}}_{i}-{{y}_{p}}_{i})}^{2}}$$	
*Coefficient of determination (R* ^*2*^ *)*	(0, 1) 1
$$\:{{R}^{2}=\left(\frac{{\sum\:}_{\text{i}=1}^{N}\left({{y}_{m}}_{i}-\stackrel{-}{{y}_{m}}\right)\left({{y}_{p}}_{i}-\stackrel{-}{{y}_{p}}\right)}{\sqrt{\left[{\sum\:}_{\text{i}=1}^{N}{\left({{y}_{m}}_{i}-\stackrel{-}{{y}_{m}}\right)}^{2}\right]\left[{\sum\:}_{\text{i}=1}^{N}{\left({{y}_{p}}_{i}-\stackrel{-}{{y}_{p}}\right)}^{2}\right]}}\right)}^{2}$$	
*Nash-Sutcliffe efficiency (NSE)*	($\:-\infty\:$, 1) 1
$$\:NSE=1-\frac{{\sum\:}_{\text{i}=1}^{N}{\left({{y}_{m}}_{i}-{{y}_{p}}_{i}\right)}^{2}}{{\sum\:}_{\text{i}=1}^{N}{\left({{y}_{m}}_{i}-\stackrel{-}{{y}_{m}}\right)}^{2}}$$	
*Performance index (PI)*	(0, 1) 0
$$\:PI=\frac{\text{R}\text{M}\text{S}\text{E}}{\stackrel{-}{{y}_{m}}\:(1+\text{R})}$$	
*Variance Accounted For (VAF)*	(0, 100) 100%
$$\:VAF=\left(1-\:\frac{var({y}_{m}-\:{y}_{p})}{var\left({y}_{m}\right)}\right)\:\times\:100$$	
*Objective function (OBJ)*	(0, $\:+\infty\:$) 0
$$\:OBJ=\left(\frac{{N}_{Tr}}{N}.\frac{{RMSE}_{Tr}+\:{MAE}_{Tr}}{{R}_{Tr}^{2}+1}\right)+\:\left(\frac{{N}_{Va}}{N}.\frac{{RMSE}_{Va}+\:{MAE}_{Va}}{{R}_{Va}^{2}+1}\right)+\left(\frac{{N}_{Te}}{N}.\frac{{RMSE}_{Te}+\:{MAE}_{Te}}{{R}_{Te}^{2}+1}\right)$$	

The number of data related to *N* = total, *N*_*Tr*_ = training, *N*_*Va*_ = validating, and *N*_*Te*_ = testing sets. $\:{{y}_{m}}_{i}$ and $\:{{y}_{p}}_{i}$ = The measured and predicted CF of the *i*th data, respectively. $\:\stackrel{-}{{y}_{m}}$ and $\:\stackrel{-}{{y}_{p}}$ = The average of total $\:{{y}_{m}}_{i}$ and $\:{{y}_{p}}_{i}$, respectively.

### Cross-validation

To evaluate the model’s performance more thoroughly, a cross-validation method is applied. This approach involves performing several assessments using *k*-fold cross-validation, which helps mitigate overfitting problems related to small datasets and biases in the training set. For this purpose, the training dataset is split into ten equal segments. In each round, nine segments are trained and the tenth is evaluated. This trend is repeated for all ten segments, and the results are averaged to provide an overall performance assessment.

## Results and discussion

### Hyperparameter tuning of models

**Fig. 10:**
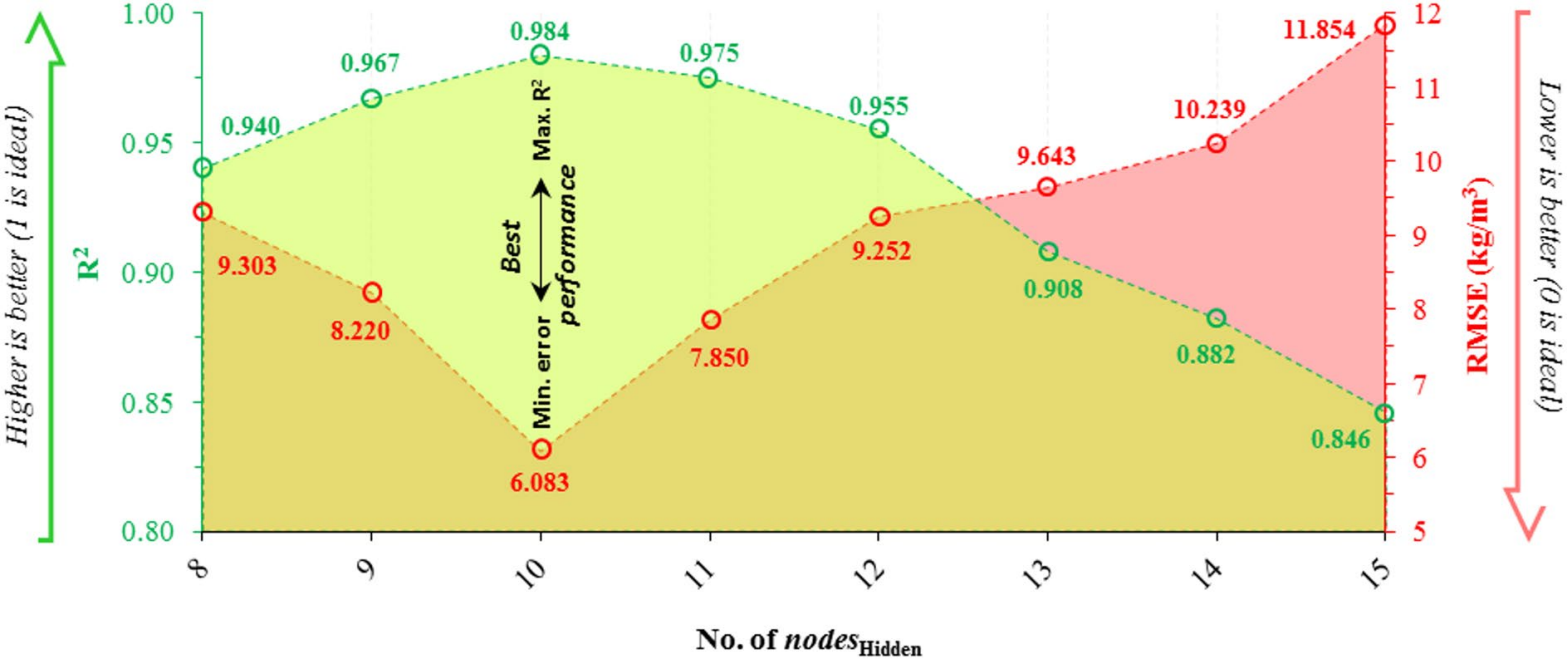
Assessing the models with different nodes_Hidden_ to determine the final structure model.

The current architecture of the model follows a *25*_Input_-*nodes*_Hidden_-*1*_Output_ structure, according to the number of input and output variables. To complete the model configuration and define *nodes*_Hidden_, the quantity of nodes in the hidden layer is evaluated to enhance performance while minimizing error. For a fair comparison, the same data sets were utilized to implement each model. In Fig. 10, the performance of different architectures is evaluated using R^2^ and RMSE values. The best performance is achieved by the architecture with R^2^ values close to 1 and RMSE values close to 0. As shown in Fig. 10, the architecture with 10 *nodes*_Hidden_ outperforms the others, achieving the highest R^2^ value of 0.984 and the lowest RMSE of 6.083 kg/m^3^. Therefore, the *25*_Input_-*10*_Hidden_-*1*_Output_ configuration was deemed the most appropriate as the final architectural structure.

**Fig. 11:**
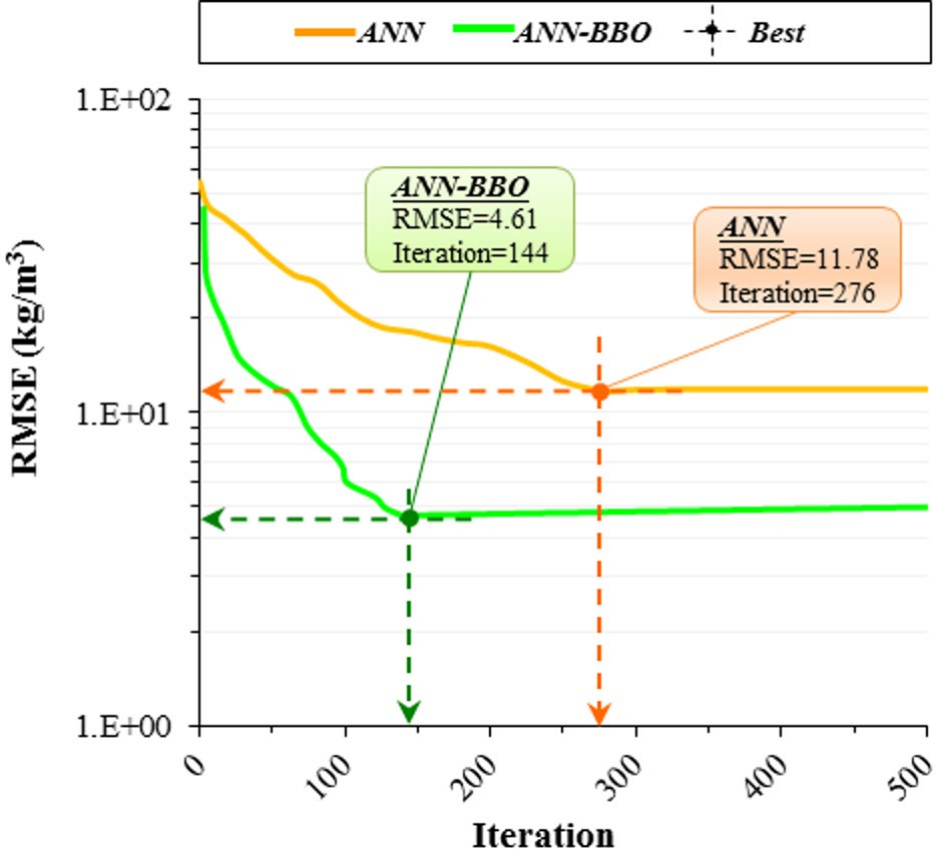
Convergence curves of models.

Figure 11 illustrates the convergence performance of the two models used in this study: ANN and ANN-BBO. As shown, the ANN model achieved its best performance with an RMSE of 11.78 after 276 iterations, while the ANN-BBO model reached an RMSE of 4.61 in just 144 iterations. These results indicate that the BBO method not only converges significantly faster but also outperforms the single ANN model in achieving a lower error with fewer iterations.

Table 4 summarizes the details of selected parameter settings to present the information more clearly.

**Table 4:** Details of selected parameter settings for ANN and BBO models.

Model	Parameter		Setting
ANN	Data dividing (%-No. of data)	Training	70%–86
		Validating	15%–18
		Testing	15%–18
	No. of hidden layer/nodes		1/10
	Learning algorithm		Levenberg–Marquardt
	Transfer function		Hyperbolic tangent
	Loss function		RMSE
	No. of iterations		500
BBO	Population size		100
	Max immigration and emigration rate		1
	Habitat modification		1
	Mutation probability		0.005
	No. of generations (iterations)		500

### Computational complexity

Evaluating computational complexity as a key performance metric helps in assessing the actual computational demands of the proposed models. To illustrate this aspect, Big-O notation is employed to express the worst-case efficiency of a model, which is influenced by both the dataset size and the model’s structure^63^. Therefore, the total computational complexity for the ANN and ANN-BBO models can be expressed as follows:

3 $$O_{ANN}=O(g(O_{ANN}))$$

4 $$O_{ANN-BBO}=O(g(O_{ANN}+O_{BBO}))$$

where: *g* = Max. No. of generations (iterations).

To this end, in each model, the overall complexity can be achieved by summing the complexities in each part of the model. The computational complexity of a single ANN model, *O*_*ANN*_ is calculated as follows:

5 $$O_{ANN}=O(g(i.h+h.o))$$

where: *i* = No. of training dataset, *h* = No. of hidden layer nodes, *o* = No. of outputs.

For the BBO model, the computational complexity includes the migration and mutation operators, which are *O*(*mn*^*2*^) and *O*(*nm*), respectively, in the worst case. In total, the computational complexity of BBO is calculated as follows:

6 $$O_{BBO}=O(g(mn^{2}+mn))$$

where: *m* = No. of habitants, *n* = No. of habitats.

### Scatter plot of predicted and measured results

**Fig. 12:**
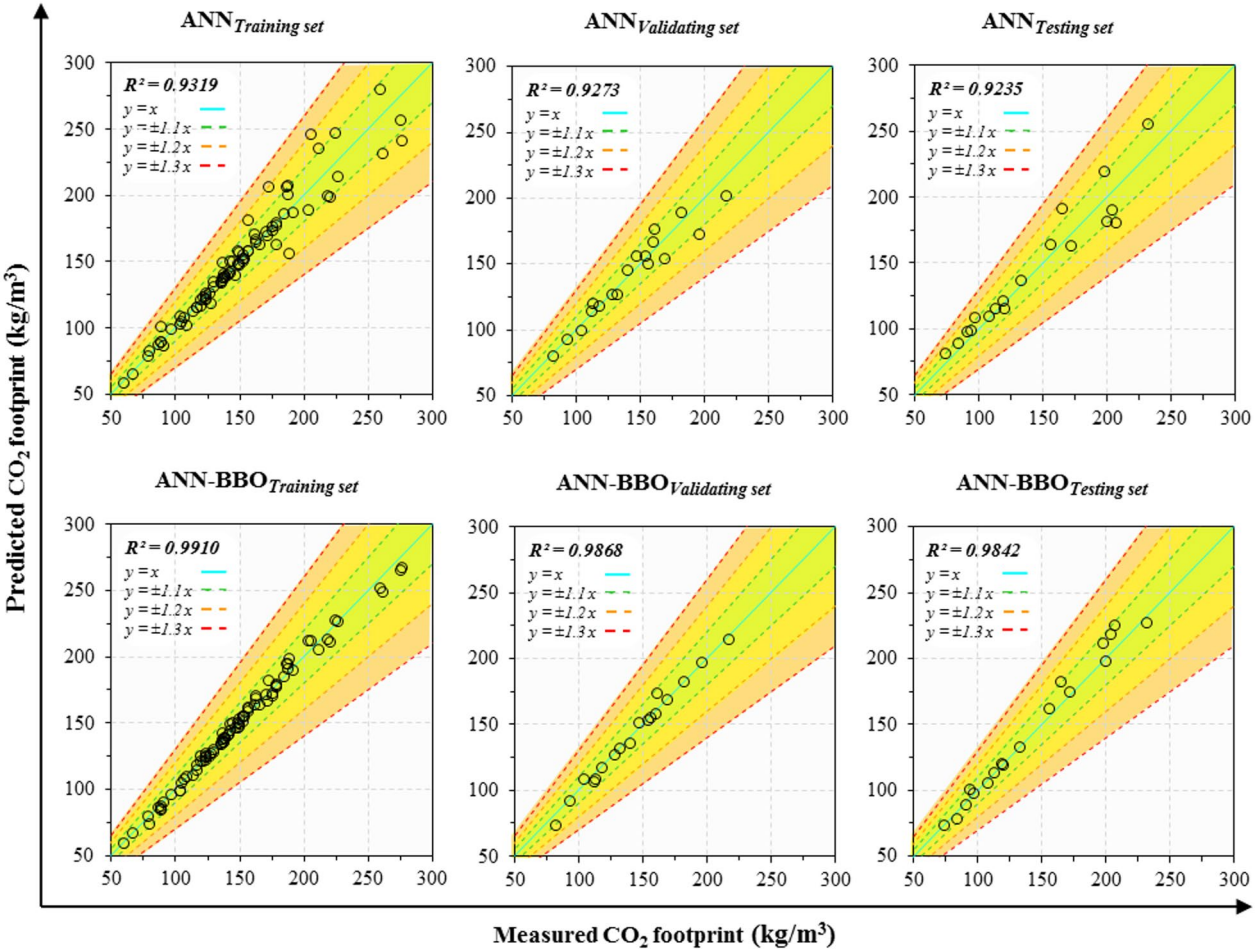
Scatter plot of predicted CO_2_ footprint vs. measured CO_2_ footprint values.

In Fig. 12, the scatter plots depict the correlation between the predicted and measured CF values across three datasets for the two proposed ANN and ANN-BBO models. The solid blue line (*y* = *x*) represents the best-fitting regression line. The predicted outcomes of the ideal model should coincide with this line, demonstrating that the predicted values perfectly match the measured data (R^2^ = 1) with no error. In addition, the dashed green, orange, and red lines on both sides specify confidence intervals of ± 10%, ± 20%, and ± 30%, respectively. As can be seen from these figures, the data points achieved by the proposed ANN-BBO are closer to the *y* = *x* line in all three data sets compared to those of the ANN. The R^2^ values obtained by the ANN-BBO for the training, validating, and testing sets are 0.9910, 0.9868, and 0.9842, respectively, while the corresponding values for the ANN are 0.9319, 0.9273, and 0.9235. Accordingly, the proposed ANN-BBO demonstrates a stronger performance correlation, highlighting its advantage over the single ANN.

### Models’ error analysis

**Fig. 13:**
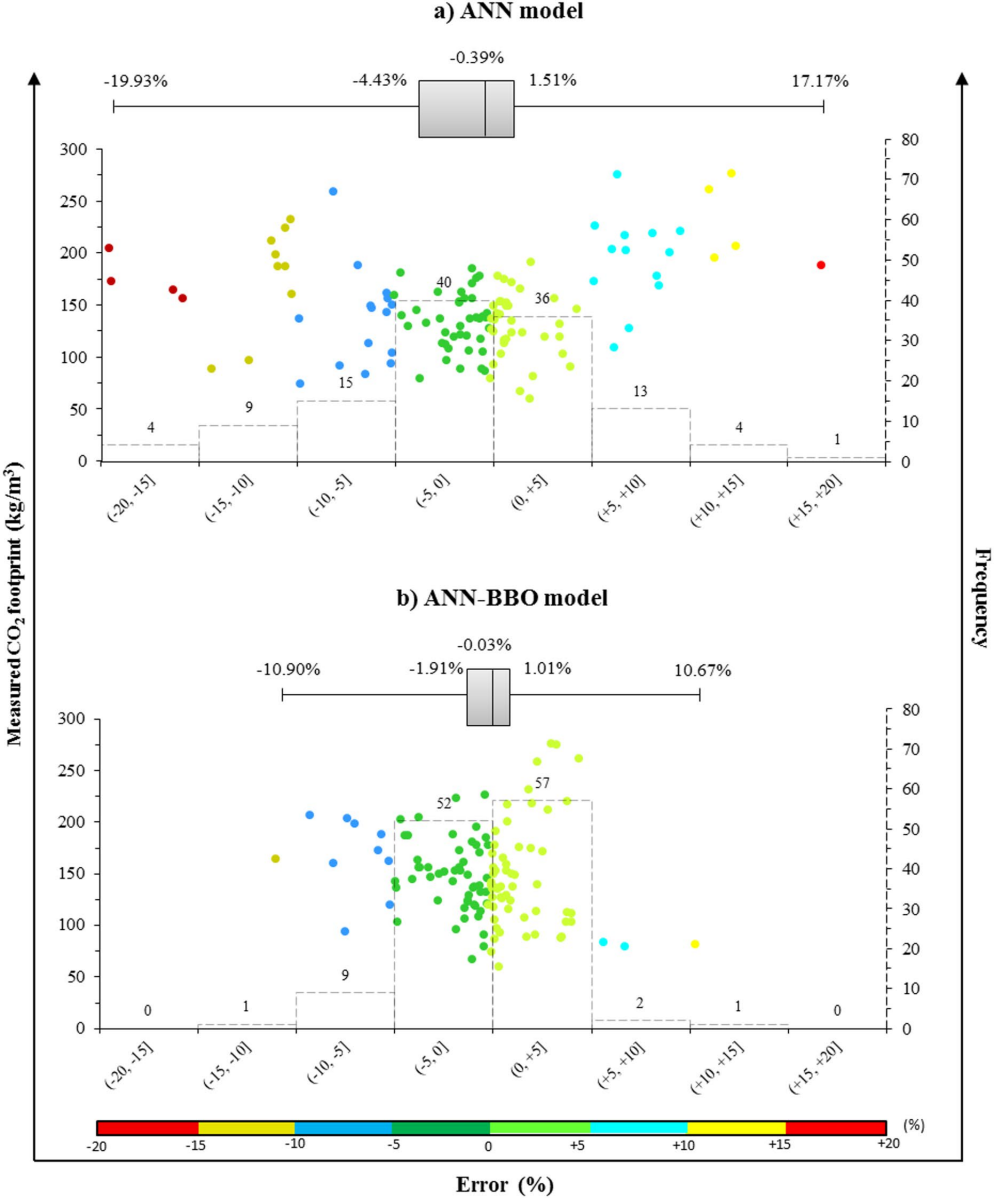
Distribution map and histogram plot of prediction error of the proposed models.

Examining the error distribution of the models can provide a deeper understanding of their performance. Figure 13 depicts the distribution map and histogram of the prediction error for two proposed models of (a) ANN and (b) ANN-BBO. Scrutinizing the differences between predicted and measured CF error values reveals that the ANN-BBO estimated the CF of GGBFS-based GPC with a smaller margin of error than the ANN. For example, when examining the frequency of error intervals, 62% of the ANN predictions (76 out of 122 data points) fall within a 5% margin of error, specifically in the range of (−5%, + 5%]. In contrast, the ANN-BBO model achieved 89% accuracy (109 out of 122 data points), reflecting a 27% improvement in prediction accuracy with the hybrid model. This demonstrates the enhanced reliability of the ANN-BBO for more accurate predictions.

**Fig. 14:**
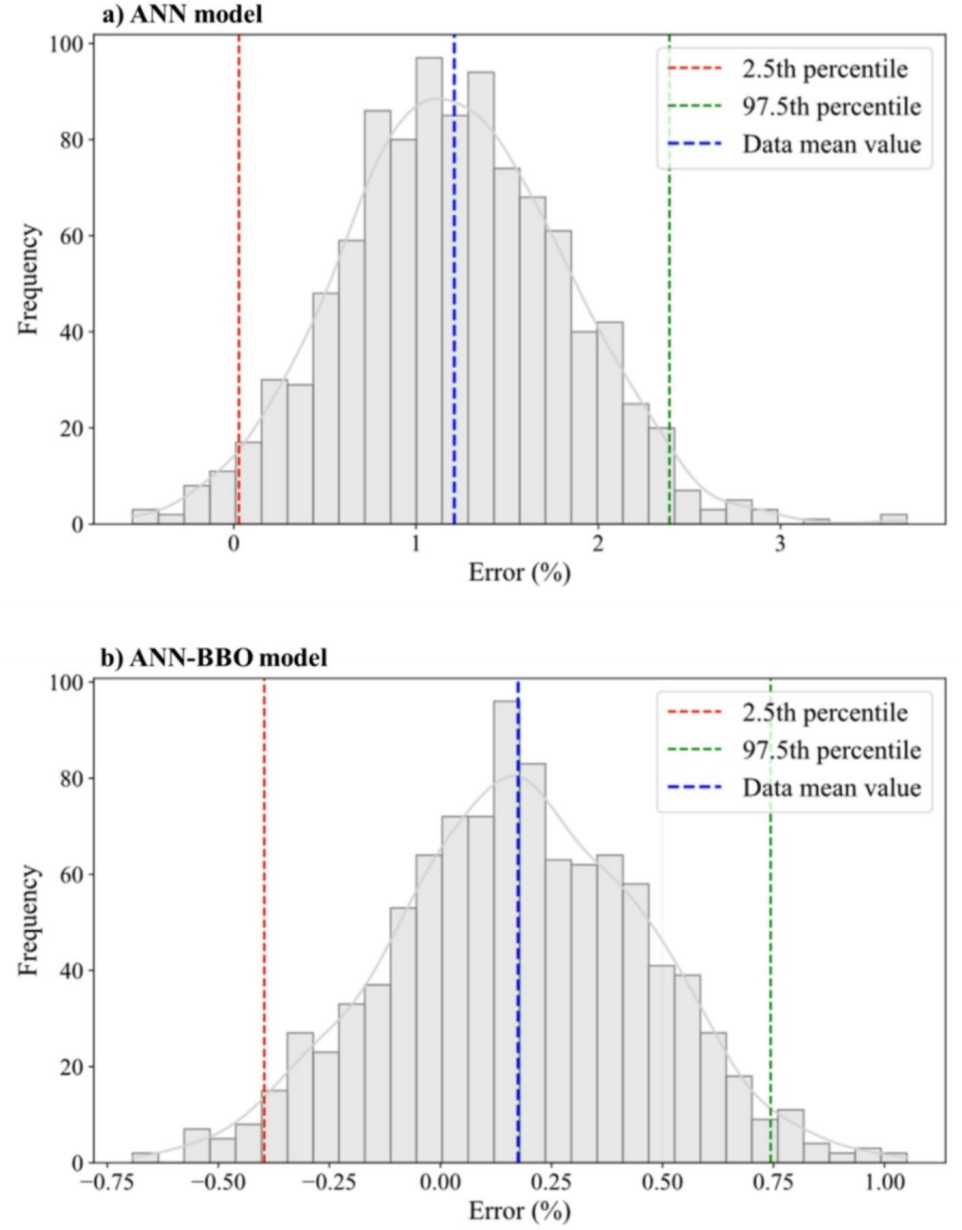
Bootstrap distribution of the proposed models.

Figure 14 illustrates the bootstrap distributions of percentage error for the two evaluated models: (a) ANN and (b) ANN-BBO, enabling a comparative assessment of their predictive performance and reliability. The ANN model yields a mean error of approximately 1.20%, indicating a consistent overestimation trend and reflecting a considerable bias in its predictions. In contrast, the ANN-BBO model achieves a substantially lower mean error of about 0.17%, representing an improvement of approximately 85% in predictive accuracy compared to the ANN model. Furthermore, the 95% confidence interval (CI) (spanning the 2.5th to 97.5th percentiles) for the ANN model covers a wider range (2.36%) than that of the ANN-BBO model (1.14%), indicating that the proposed hybrid model also offers a 52% reduction in prediction uncertainty. Additionally, the error distribution of the ANN model is positively skewed, highlighting a systematic tendency toward overprediction, whereas the ANN-BBO model displays a more symmetric and centered distribution around zero. A summary of these results is presented in Table 5. These findings collectively confirm that the proposed hybrid ANN-BBO model outperforms the single ANN in terms of both accuracy and consistency, making it a more robust and generalizable predictive model.

**Table 5:** Summary of the bootstrap-based performance for the models.

Metric	ANN model	ANN-BBO model	Better model
Mean error (%)	1.20	0.17	ANN-BBO
CI width (95%) (%)	2.36	1.14	ANN-BBO
2.5th percentile (%)	0.02	−0.40	-
97.5th percentile (%)	2.38	0.74	-
Distribution shape	Right-skewed	Symmetric	ANN-BBO
Bias (Error from 0%)	High	Low	ANN-BBO

### Evaluation of performance metrics

**Fig. 15:**
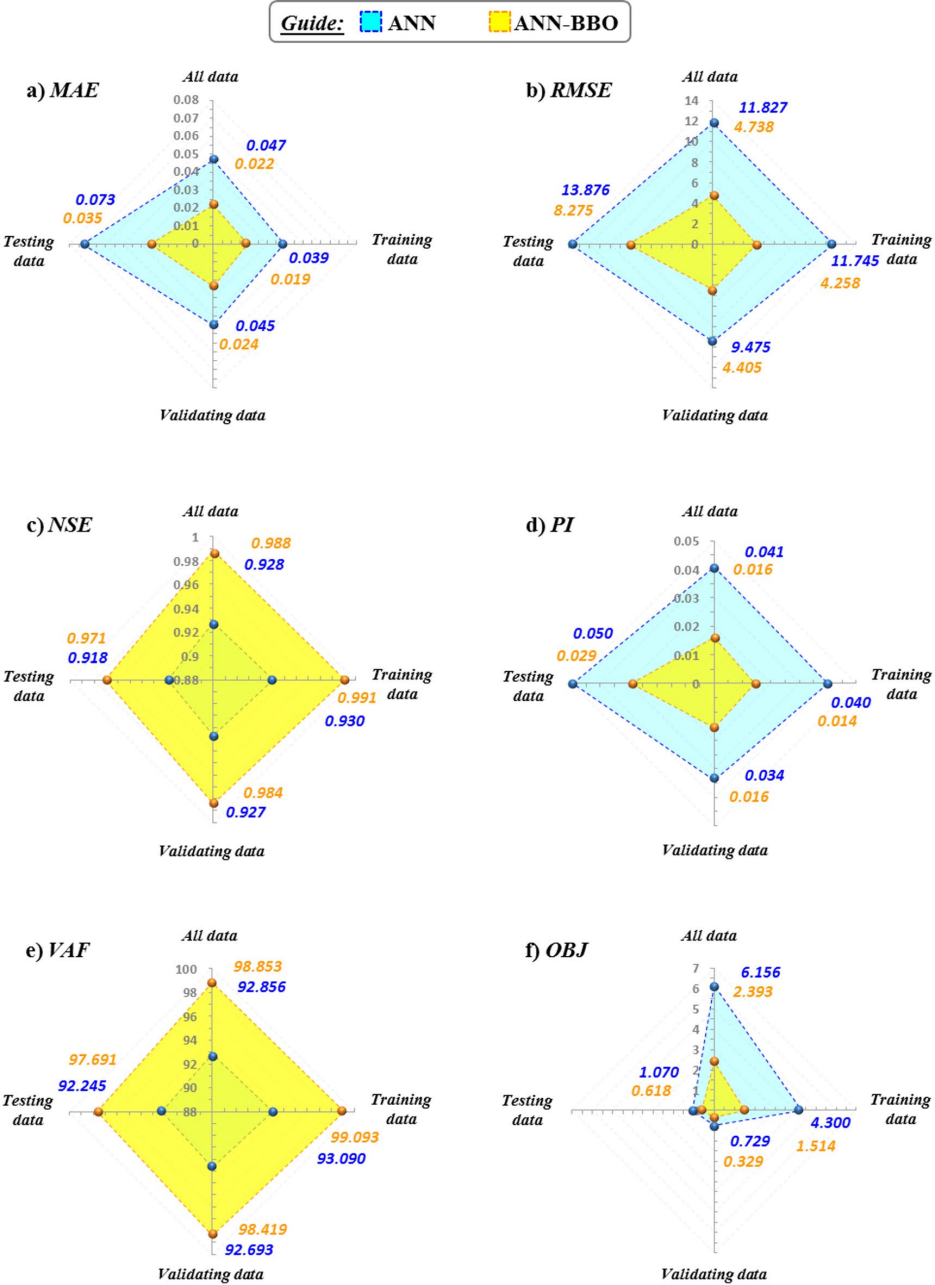
A comparative presentation of the performance metrics for the proposed models across three data sets.

Figure 15 visualizes the performance metrics of the two proposed models in the form of radar charts utilizing six statistical measures: MAE (a), RMSE (b), NSE (c), PI (d), VAF (e), and OBJ (f). Understanding the range of excellent performance of these metrics allows for better evaluation. It is crucial to highlight that the ideal performance for MAE, RMSE, PI, and OBJ is achieved when their values approach zero, whereas the ideal values for NSE and VAF are close to 1 and 100%, respectively. As observed in the figure, the ANN-BBO exhibits lower errors for both MAE and RMSE error metrics across all three data sets. For instance, a comparison of the results from these two metrics for the training data set reveals a decrease of approximately 51% in MAE and 63% in RMSE for the ANN-BBO compared to the ANN. A similar pattern can be seen in the PI values. The results show that the ANN-BBO is associated with higher NSE and VAF values compared to the ANN in three datasets, further highlighting its superior performance. Regarding the OBJ metric, it serves as a combined error metric, indicating that the ANN-BBO outperforms the single ANN by 61% across all data (see Fig. 15f). A summary of the obtained evaluation metrics values ​​is tabulated in Table 6. The performance metrics results are consistent with the findings of the previous subsections, confirming the effectiveness of the proposed hybrid model in estimating the CF of GGBFS-based GPC.

**Table 6:** Tabulation of the obtained performance metrics values.

Model	Data set	Metrics					
		MAE (kg/m^3^)	RMSE (kg/m^3^)	NSE	PI	VAF (%)	OBJ (kg/m^3^)
ANN	Training	0.039	11.745	0.930	0.040	93.090	4.300
	Validating	0.045	9.475	0.927	0.034	92.693	0.729
	Testing	0.073	13.876	0.918	0.050	92.245	1.070
	All	0.047	11.827	0.928	0.041	92.856	6.156
ANN-BBO	Training	0.019	4.258	0.991	0.014	99.093	1.514
	Validating	0.024	4.405	0.984	0.016	98.419	0.329
	Testing	0.035	8.275	0.971	0.029	97.691	0.618
	All	0.022	4.738	0.988	0.016	98.853	2.393

### Cross-validation analysis

**Fig. 16:**
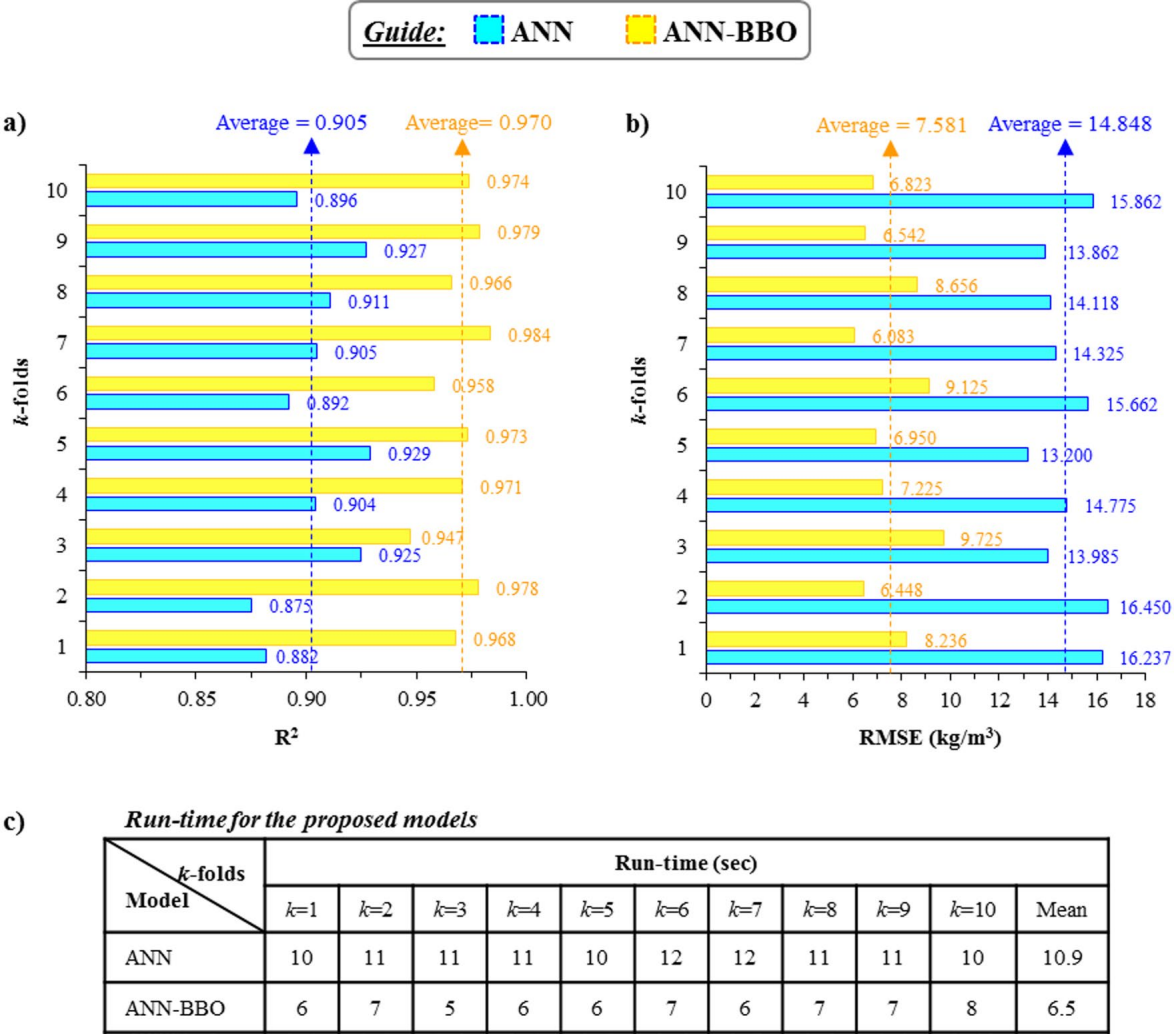
Evaluation of the cross-validation analysis: (**a**) R^2^, (**b**) RMSE, and (**c**) run-time.

To assess the reliability and generalization capabilities of the proposed models on new datasets, *k*-fold cross-validation is implemented. In this context, Fig. 16 illustrates the R^2^ and RMSE performance metrics of the models based on the results from a ten-fold cross-validation analysis. The dotted lines represent the mean performance metric values across all folds. As illustrated in Fig. 16a, the R^2^ values of the ANN ranged from 0.875 to 0.929 across the analyzed 10-fold. In comparison, the ANN-BBO demonstrated superior performance, achieving R^2^ values between 0.947 and 0.984. On average, the R^2^ values obtained by the ANN and ANN-BBO were 0.905 and 0.970, respectively. Furthermore, the ANN-BBO achieved an average RMSE of 7.581, representing a 49% reduction in error compared to the ANN model’s average RMSE of 14.848 (see Fig. 16b). Additionally, the runtime of the models shows that ANN-BBO completed its execution in a shorter time, with an average of 6.5 s, compared to ANN, which had an average runtime of 10.9 s. As a result, the hybrid model demonstrates faster execution than the single model (see Fig. 16c). The findings of this analysis suggest that the proposed ANN-BBO excels in its ability to generalize to new datasets.

### Sensitivity analysis

To determine the impact of each input variable on the CF of GGBFS-based GPC, a relevancy factor (*RF*) analysis was performed. Throughout this analysis, the *RF* value of each variable represents the intensity of its influence on the CF of GGBFS-based GPC, while the sign of the *RF* denotes whether the input variable has a positive or negative effect. The *RF* equation is expressed as follows:

7 $$\:{RF}_{\left({x}_{i\:}\right)\:}=\frac{\sum\:_{N}{(x}_{i,N}-\:\stackrel{-}{{x}_{i}}\left)\right({y}_{N,p}-\stackrel{-}{{y}_{p}})}{\sqrt{{\sum\:_{N}{(x}_{i,N}-\:\stackrel{-}{{x}_{i}})}^{2}{\sum\:_{N}{(y}_{N,p}-\:\stackrel{-}{{y}_{p}})}^{2}}}\:$$

where *x*_*i*_ and *x*_*i, N*_ are the *i*th input variable and the its value for the *N*th data point. $\:\stackrel{-}{{x}_{i}}$ is the average of the values of the input variable with index *i*. *y*_*N, p*_ and $\:\stackrel{-}{{y}_{p}}$ are the predicted CF of the *N*th data point and the average of the values of the predicted CF.

**Fig. 17:**
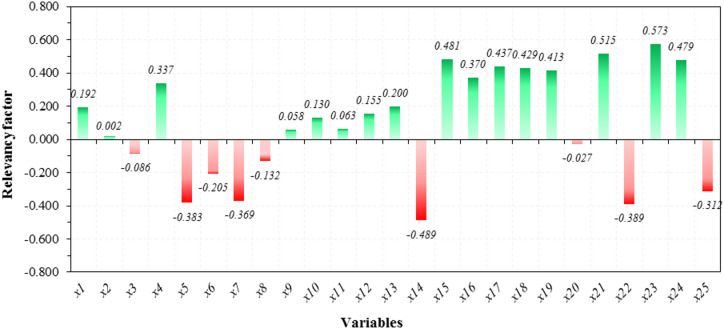
Relevance factors obtained for the top-performing model.

Figure 17 presents the analysis results based on the CO_2_ outcomes derived from the best-performing model (ANN-BBO). As shown in the figure, the most critical positive variable in controlling the CF of GGBFS-based GPC is the *x*_23_ (superplasticizer), with an *RF* value of 0.573. Following *x*_23_, the second, third, and fourth most crucial input variables are *x*_21_ (NaOH (Dry)), *x*_15_ (fine aggregate), and *x*_24_ (initial curing temperature), respectively. This means that increasing these variables in the mix design contributes to an increase in the CF. In contrast, the *x*_14_ (coarse aggregate) exhibits a strong inverse relationship. Also, the interdependence between the correlated variables *x*_15_ (fine aggregate) and *x*_14_ (coarse aggregate) affects binder demand and overall concrete properties, which in turn influences the required amounts of superplasticizer (*x*_23_) and NaOH (*x*_21_) geopolymer precursor—a major contributor to CO_2_ emissions^64^. An increase in coarse aggregate content can reduce binder demand per unit volume, potentially lowering CO_2_ emissions associated with geopolymer precursor production. Conversely, excessive coarse aggregate can compromise workability, leading to greater fine aggregate usage or the addition of admixtures, thereby increasing emissions^65^. In conclusion, CO_2_ emissions are highly sensitive to the fine-to-coarse aggregate ratio, underscoring the need to carefully manage their interdependence in mix design to minimize environmental impacts.

**Fig. 18:**
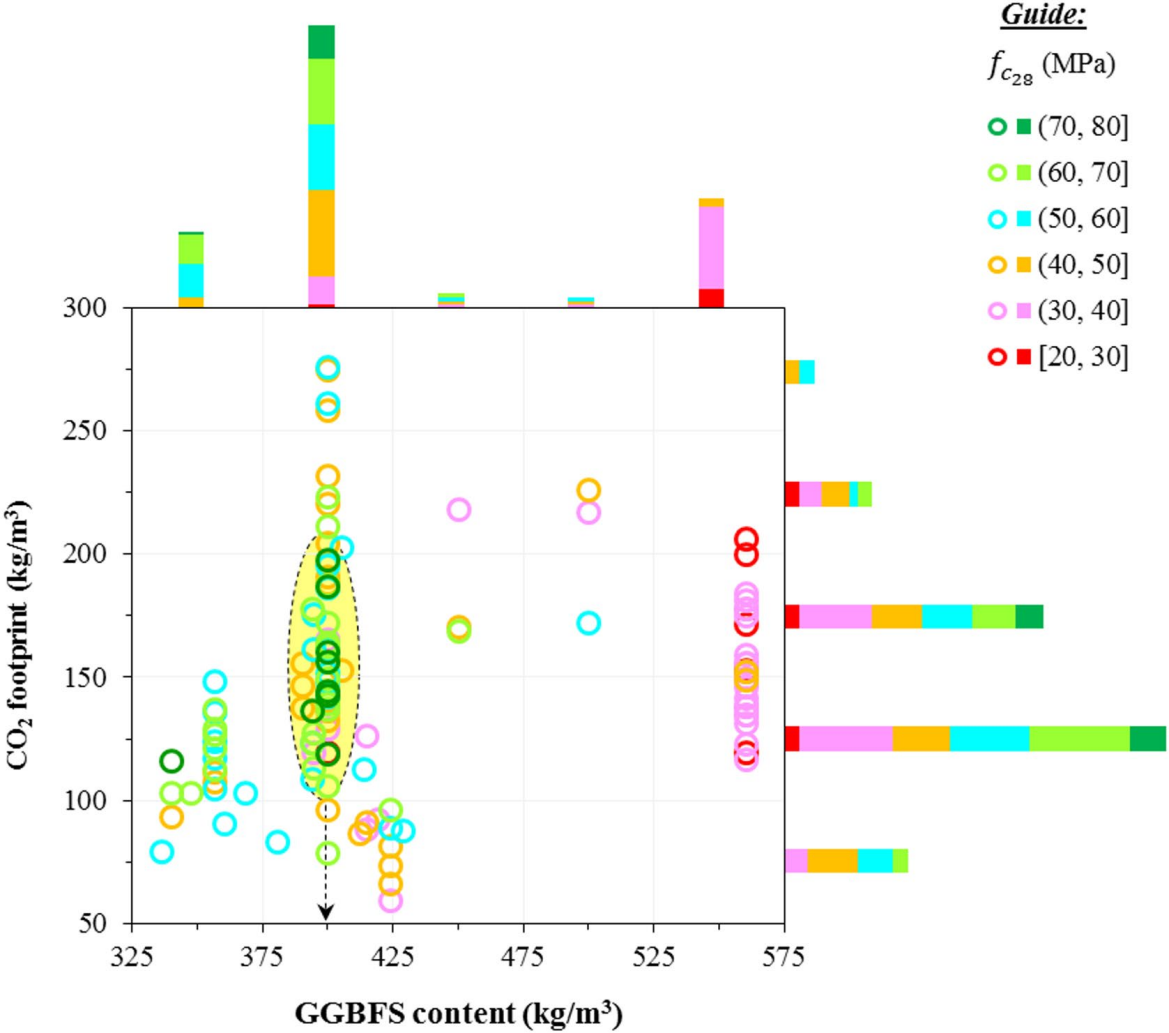
Relationship between CO_2_ footprint vs. GGBFS content and $\:{f}_{{c}_{28}}$ in the GGBFS-based GPC.

Further analysis has been conducted on the CF and GGBFS content, focusing on the compressive strength (*f*_c28_) of mix designs. As shown in Fig. 18, the CF of the mix designs typically remains below 200 kg/m^3^. However, the GGBFS content influences the *f*_c28_. The results indicate that GPCs with higher GGBFS content tend to have lower *f*_c28_, while the optimum GGBFS content for achieving the highest *f*_c28_ is 400 kg/m^3^. This optimal amount of GGBFS results in a *f*_c28_ of up to 80 MPa with a CF of 100–200 kg/m^3^.

### Comparing the top-performing model with literature models

**Fig. 19:**
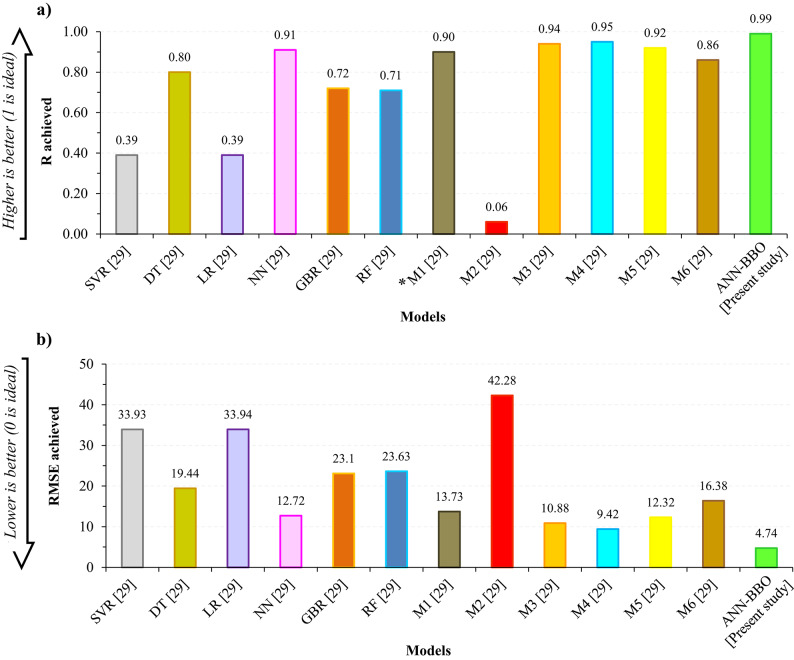
Comparative analysis of the top-performing model and previous study models. Note: SVR= Support vector regression; *DT*= Decision tree; *LR*= Linear regression; *NN*= Neural network; GBR= Gradient boosting regression; RF= Random forest. *The stacking ensemble models defined according to Table 2 of Ref. [29].

The ultimate and most important step in AI research is undoubtedly validating the proposed model by benchmarking it against other AI methods to demonstrate its superiority and effectiveness. For a fair comparison, it is crucial to ensure that the subject of study and the model’s output are consistent. Therefore, a comparative analysis has been conducted to assess the top-performing model of this study against a recent related work^29^, which employed the same dataset to estimate the CF of GGBFS-based GPC using various AI techniques. Since in^29^ details on the hyperparameter tuning of optimization methods used and the run-time performance of the models are not reported, it limits direct comparison in these aspects. However, the performance of the models was evaluated using two reported metrics, R and RMSE, as shown in Fig. 19, to assess their accuracy. A comparison of the results shows that the proposed ANN-BBO performed better than the models found in the literature, achieving a higher R-value and a lower RMSE. This outcome demonstrates the superior performance and efficiency of the proposed ANN-BBO over those in the recent study for predicting the CF of GGBFS-based GPC.

## Limitations and implications

As we progress toward utilizing AI to predict the CF of GGBFS-based GPC production, several challenges and limitations remain that need to be addressed in future work to pave the research way. Data quality and availability present major challenges, as obtaining experimental data is typically expensive and time-intensive. Consequently, the resulting datasets are often limited in size and may fail to encompass the full range of GGBFS-based GPC designs. In this regard, the authors acknowledge that to prevent potential model drift, bias-variance tradeoff, and model generalizability (important concerns in production systems where real-world data evolves) periodic retraining of the model with newly arrived data is planned. Furthermore, the simplified model for calculating the CF does not account for the contribution of material transportation from the production/extraction site to the batching plant due to a lack of relevant data. Besides, another challenge lies in the disparity between mass production setups and the controlled conditions of laboratory production. Although laboratory equipment has made significant advancements, validating experimental results remains a costly and time-intensive bottleneck due to the numerous variables involved in the CF. Additionally, there is still insufficient AI research on this subject to make meaningful comparisons. For example, Al-Fakih et al.^29^ developed AI models in their study but did not compare their performance with other studies.

## Conclusions and future directions

Since the construction industry plays a major role in global carbon emissions, adopting sustainable production practices by using/reusing industrial byproducts and waste materials is an effective strategy to promote low-carbon development. In this regard, this study deals with the investigation of the CO_2_ footprint of GGBFS-based GPC. Given that laboratory experiments are often costly, time-intensive, and necessitate repeated testing to assess variations in material ratios and conditions, this study aims to develop a precise and dependable AI-based framework for predicting the CO_2_ footprint of GGBFS-based GPC. This was achieved by leveraging a novel hybrid ANN-BBO model. In this context, the models’ performance was assessed using evaluation metrics, while their reliability and generalizability were analyzed through cross-validation. The influence of variables on model development was examined using the relevancy factor. Lastly, the proposed model’s superiority was determined by comparing it with the literature model. A concise summary of the key findings of the research is provided below.

1. By scrutinizing the performance of the proposed models using statistical metrics, it was found that the proposed ANN-BBO outperformed the single ANN. In this regard, the OBJ metric, as a combined metric consisting of MAE, RMSE, and R^2^, showed superior performance close to zero for the ANN-BBO with a value of 2.393 compared to the value of 6.156 obtained for the single ANN.
2. The distribution map and histogram of the prediction error showed that 62% of the ANN predictions fell within a 5% margin of error, whereas the ANN-BBO had 89% within the same margin, demonstrating a 27% improvement in prediction accuracy with the proposed hybrid model.
3. Cross-validation analysis demonstrated the superiority of the proposed hybrid model over the ANN in effectively addressing reliability and generalizability.
4. Sensitivity analysis revealed that the key positive and negative variables affecting the CO_2_ footprint were the superplasticizer and coarse aggregate, respectively. Furthermore, the optimum GGBFS content for GGBFS-based GPC production to achieve maximum compressive strength is 400 kg/m^3^.
5. To the authors’ knowledge, only one previous study has addressed the prediction of the CO_2_ footprint of GGBFS-based geopolymer concrete using AI approaches. A comparison with that study, which employed several AI models, indicated that the hybrid ANN-BBO model proposed in this research outperformed the earlier models. Nonetheless, the need for broader benchmarking remains as areas for ongoing development.

By accurately predicting the CO_2_ footprint associated with geopolymer concrete mixtures, the proposed model can support more informed material selection and mix design optimization, contributing to the development of low-carbon alternatives in the construction industry. The identification of key influencing variables, also provides actionable insights for practitioners aiming to minimize emissions without compromising mechanical performance. Future research will include the experimental validation of the proposed model using independently designed laboratory mixes. This will help assess the model’s predictive performance under controlled conditions and improve its generalizability beyond the literature-based dataset. Also, future studies should prioritize feature importance analysis that ensures the role of real variables while minimizing bias from highly correlated variables. Moreover, enhancing the modeling and calculation processes of the variables involved, especially in aspects like material transportation, is crucial for attaining a more comprehensive evaluation of the environmental impacts associated with GGBFS-based GPC production. Furthermore, the predictive capabilities of the model present opportunities for integration into eco-labeling schemes, greenhouse gas (GHG) accounting frameworks, and digital tools for sustainable infrastructure planning. A future research direction could target more detailed life cycle assessment (LCA) that considers region-specific emission factors, transportation-related emissions, and energy source variations to improve the accuracy and real-world relevance of CO_2_ footprint estimates. As governments and industries increasingly prioritize carbon transparency, such data-driven approaches can serve as valuable enablers of environmental compliance and green certification. Future work could extend this model to broader datasets, include additional environmental metrics, and explore its adaptability to other sustainable binder systems.

## Supplementary Information

Below is the link to the electronic supplementary material.

Supplementary Material 1

## Data Availability

The database used in this study is available in the supplementary file.

## Abbreviations

AI: Artificial intelligence
Al_2_O_3_: Alumina
ANN: Artificial neural network
BBO: Biogeography-based optimization
C: Coke
CaCO_3_: Limestone
CaO: Calcium oxide
CF: CO_2_ footprint
CI: Confidence interval
Fe_2_O_3_: Iron ore
f_c28_: Compressive strength at 28 days
GGBFS: Ground granulated blast-furnace slag
GHG: Greenhouse gas
GPC: Geopolymer concrete
HSI: Habitat suitability index
LCA: Life cycle assessment
MAE: Mean absolute error
N: Number of total data
N_Te_: Number of testing data
N_Tr_: Number of training data
N_Vd_: Number of validating data
NaOH: Sodium hydroxide
Na_2_SiO_3_: Sodium silicate
NSE: Nash-Sutcliffe efficiency
nodes_Hidden_: Number of hidden layer nodes
OBJ: Objective function
OPC: Ordinary Portland cement
PI: Performance index
R^2^: Coefficient of determination
r: Pearson correlation coefficient
RF: Relevancy factor
RMSE: Root mean squared error
SCMs: Supplementary cementitious materials
SDGs: Sustainable development goals
SIV: Suitable index variables
SiO_2_: Silica
VAF: Variance accounted for
y_m_: Measured value
y_p_: Predicted value
